# Secreted tyrosine sulfated-eIF5A mediates oxidative stress-induced apoptosis

**DOI:** 10.1038/srep13737

**Published:** 2015-09-08

**Authors:** Yoshinori Seko, Tsutomu Fujimura, Takako Yao, Hikari Taka, Reiko Mineki, Ko Okumura, Kimie Murayama

**Affiliations:** 1 Department of Cardiovascular Medicine, The Institute for Adult Diseases, Asahi Life Foundation, 2-2-6 Nihonbashi-Bakurocho, Chuo-ku, Tokyo 103-0002, Japan; 2 Division of Proteomics and Biomolecular Science, BioMedical Research Center, Graduate School of Medicine, Juntendo University, Bunkyo-ku, Tokyo 113-8421, Japan; 3 Department of Immunology, Graduate School of Medicine, Juntendo University, Bunkyo-ku, Tokyo 113-8421, Japan

## Abstract

Oxidative stress plays a critical role in ischemia/reperfusion-injury, atherosclerosis, and aging. It causes cell damage that leads to apoptosis via uncertain mechanisms. Because conditioned medium from cardiac myocytes subjected to hypoxia/reoxygenation induces extensive apoptosis of cardiac myocytes under normoxia, we hypothesized that a humoral factor released from the hypoxic/reoxygenated cardiac myocytes mediates apoptosis. We identified an apoptosis-inducing humoral factor in the hypoxia/reoxygenation-conditioned medium. Here, we found that eIF5A undergoes tyrosine sulfation in the *trans*-Golgi and is rapidly secreted from cardiac myocytes in response to hypoxia/reoxygenation; then, eIF5A induces apoptosis by acting as a pro-apoptotic ligand. The apoptosis of cardiac myocytes induced by hypoxia/reoxygenation or ultraviolet irradiation was suppressed by anti-eIF5A neutralizing monoclonal antibodies (mAbs) *in vitro*. Myocardial ischemia/reperfusion (but not ischemia alone) markedly increased the plasma levels of eIF5A, and treatment with anti-eIF5A neutralizing mAbs significantly reduced myocardial injury. These results identify an important, novel specific biomarker and a critical therapeutic target for oxidative stress-induced cell injury.

Oxidative stress induced by various external stresses, such as ischemia/reperfusion, ultraviolet light, and irradiation, plays a pivotal role in the pathogenesis of cell injury, which, in turn, promotes inflammation, atherosclerosis, aging, and cancer^1^,^2^,^3^. In particular, cardiac myocytes express various molecules in response to ischemia/reperfusion to adapt to or induce further cell damage, known as reperfusion injury. The threshold at which cardiac myocytes undergo apoptosis appears to be high to protect these non-dividing cells from external stresses^4^, except for reperfusion injury. Because reperfusion-induced apoptotic cell death is not induced by ischemia alone and cannot be prevented by neutrophil depletion^5^, a mechanism that is triggered by reperfusion may mediate the apoptotic signaling pathway prior to and independent of neutrophil infiltration^6^.

## Results

We analyzed intracellular signaling activity in cardiac myocytes that were subjected to hypoxia/reoxygenation^7^,^8^ *in vitro* and found that hypoxia/reoxygenation conditioned medium (RCM) activated the same signaling pathways in cardiac myocytes under normoxia (Supplementary Fig. 1) to induce apoptosis in a dose-dependent manner (Supplementary Fig. 2). This observation strongly suggested that some humoral factor was rapidly released from the cardiac myocytes in response to hypoxia/reoxygenation and induced apoptotic signaling pathways in an autocrine fashion. Whereas control-conditioned medium (CCM), supernatants of non-stimulated cardiac myocytes under normoxia (10 min), did not activate these signaling pathways nor induce apoptosis (data not shown). We previously analyzed RCM via 2-dimensional (2-D) gel electrophoresis and mass spectrometry (MS) and identified cyclophilin A as a secreted protein^9^. However, cyclophilin A appears to act as an anti-apoptotic factor.

### Reoxygenation induces eIF5A secretion

**Figure 1:**
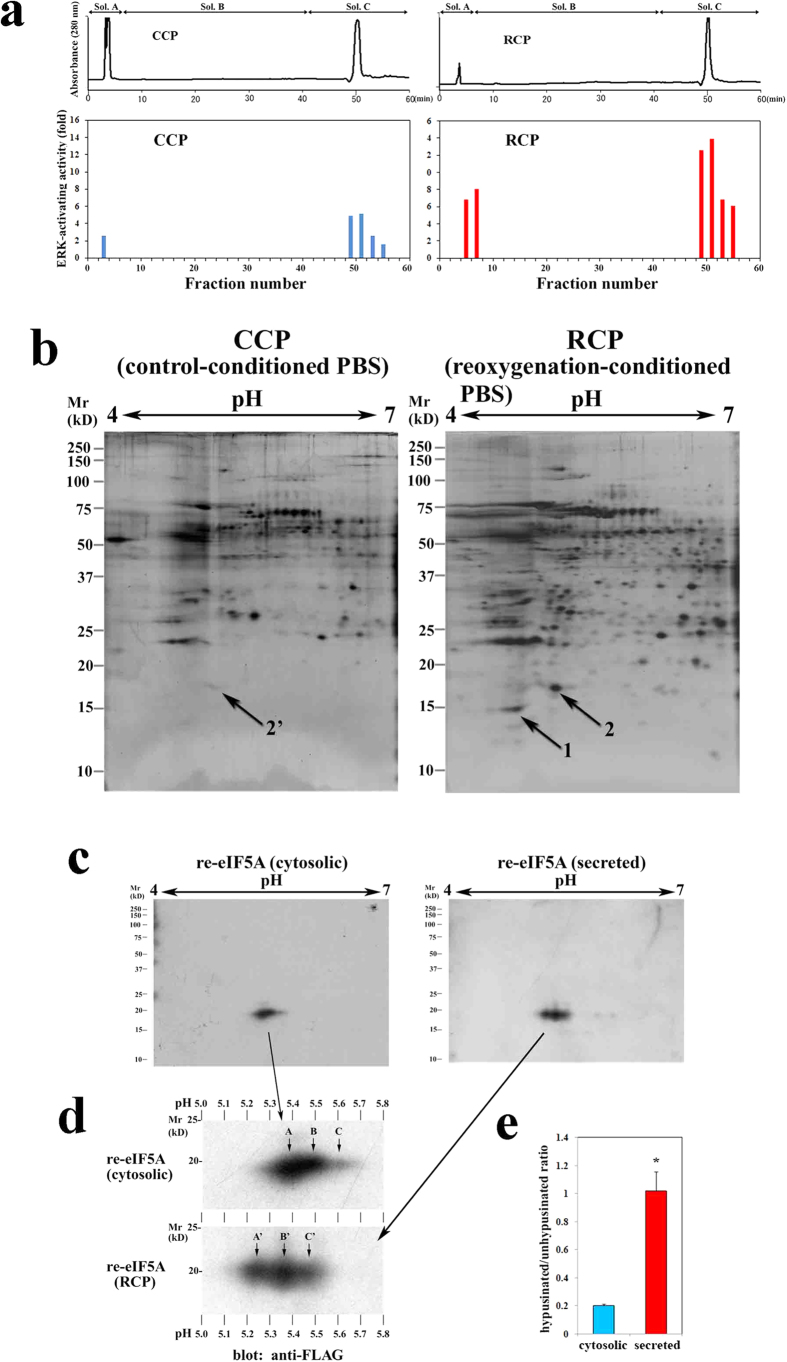
Isolation and identification of an apoptosis-inducing protein secreted from cardiac myocytes in response to hypoxia/reoxygenation. (**a**) Chromatofocusing of CCP (left panel) and RCP (Right panel) from cultured cardiac myocytes. The blue and red bars indicate the ERK-inducing effect of each fraction on the cultured cardiac myocytes. (**b**) 2-D gel electrophoresis of the ERK-activating fractions (fractions 49–52) from CCP (left panel) or RCP (right panel) followed by silver staining. The arrows indicate protein spots in the RCP (left panel) that were not detected or were very weakly detected (2′) in the CCP (left panel). (**c**) Western blot analysis of cytosolic re-eIF5A from untreated transfected cells (left panel) and re-eIF5A from the RCP (right panel); the membrane was probed with an anti-FLAG mAb and was developed via chemiluminescence using alkaline phosphatase. (**d**) Magnification of the spots shown in c (cytosolic re-eIF5A [upper panel] and re-eIF5A from RCP [lower panel]). The arrows indicate the unhypusinated (A and A’), deoxyhypusinated (B and B’), and hypusinated forms of eIF5A (C and C’). (**e**) The hypusinated/unhypusinated ratio (mean ± s.e.m.) for cytosolic and secreted re-eIF5A (n = 4 for each, *P = 0.0209) (Mann-Whitney *U*-test).

We collected and concentrated a fraction with a relative molecular mass (*M*_r_) > 10 kD from the phosphate-buffered saline (PBS) supernatants of cardiac myocytes subjected to hypoxia (60 min)/reoxygenation (10 min); this fraction was defined as reoxygenation-conditioned PBS (RCP) because it displayed extracellular signal-regulated kinase (ERK)-activating and apoptosis-inducing activities (data not shown). We also collected and concentrated PBS supernatants of non-stimulated cardiac myocytes under normoxia (10 min), designated as control-conditioned PBS (CCP). The proteins in the RCP and CCP fractions were separated via chromatofocusing (Fig. 1a). In each fraction, we monitored the induction of ERK activity, which appeared to be one of the most sensitive markers of the target factor examined, and found that fractions 49–52 (high-salt [1 M NaCl] fractions) in both the RCP and CCP groups displayed strong activity (activity, RCP > CCP; Fig. 1a) and that RCP fractions 5–8 (fractions in Solution A passed through from the column without binding) displayed much weaker activity than fractions 49–52. Then, aliquots of the fractions (49–52) from each group (corresponding to the same number of cells) were subjected to 2-D gel electrophoresis (Fig. 1b; left panel, CCP; right panel, RCP). Because the active components were not eluted by Solution B, they appeared to be acidic.

Among the spots with a low pI, spot 1 (*M*_r_ 14.4 kD and pI 4.8) and spot 2 (*M*_r_ 16.8 kD and pI 5.3), which are indicated by arrows in the RCP-based image (Fig. 1b; right panel), were not detected or were very weakly detected (spot 2′) in the CCP-based image (Fig. 1b; left panel), respectively, and appeared to have newly appeared or greatly increased in response to hypoxia/reoxygenation. LC-MS/MS analysis of protein spots 1 and 2 identified these spots as thioredoxin and eukaryotic translation initiation factor (eIF) 5A, respectively. Because thioredoxin acts as an antioxidant by reducing other proteins and potentially plays a protective role against oxidative stress, we hypothesized that eIF5A may be a candidate for the apoptosis-inducing humoral factor. eIF5A is the only known protein to contain the unique amino acid hypusine, which is formed post-translationally via a two-step enzymatic reaction^10^. eIF5A is primarily localized to the cytoplasm, where hypusinated eIF5A facilitates the translation of mRNAs that are involved in cell proliferation. Alternatively, unhypusinated eIF5A rapidly translocates from the cytoplasm to the nucleus, where it may play a role in inducing apoptosis upon death receptor (DR) stimulation^10^,^11^,^12^. However, no previous studies have reported that eIF5A performs an extracellular function as a pro-apoptotic factor.

To confirm this finding, we purified secreted and cytosolic FLAG- and His-tagged recombinant (re)-eIF5A and analyzed these re-eIF5A proteins via 2-D gel electrophoresis followed by Western blot using an anti-FLAG monoclonal antibody (mAb) (M2, Sigma) (Fig. 1c,d). Three major forms of re-eIF5A proteins were observed (Fig. 1d; upper panel, spots A, B, and C; lower panel, spots A’, B’, and C’); these forms likely represent unhypusinated, deoxyhypusinated, and hypusinated eIF5A, respectively, as reported elsewhere^12^. Figure 1d shows the relative distribution of unhypusinated:deoxyhypusinated:hypusinated eIF5A (cytosolic, 65.07%:20.96%:13.97%; secreted, 30.89%:44.54%:24.57%). We detected a significant difference in hypusination between the cytosolic and secreted forms (cytosolic, hypusinated/unhypusinated ratio = 0.203 ± 0.010 [mean ± s.e.m.]; secreted, 1.021 ± 0.135; n = 4, *P* = 0.0209) (Fig. 1e). We also detected a significant acidic shift (by approximately 0.1) in the pI values of all 3 forms of eIF5A (cytosolic, 5.37, 5.49, and 5.60, respectively; secreted, 5.25, 5.36, and 5.48, respectively) (Fig. 1d). We confirmed the shift in the pI values via 2-D Western blot analysis of a mixture of the Myc-tagged cytosolic form and FLAG-tagged secreted form of eIF5A (Supplementary Fig. 3). This experiment indicated that some structural modification occurred during secretion.

### EIF5A undergoes tyrosine sulfation

**Figure 2:**
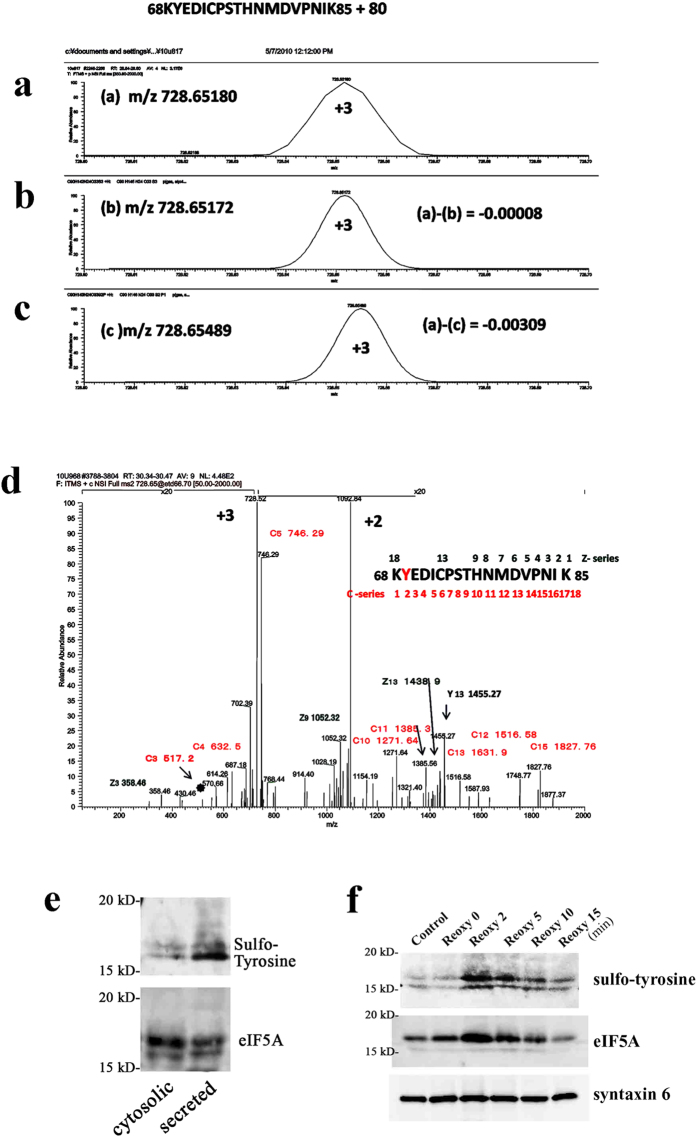
Identification of the structural modifications present in the secreted form of eIF5A. (**a**–**c**) Extracted ion chromatogram of the +3 charge state of the apparent tryptic peptide of residues 68–85 (KYEDICPSTHNMDVPNIK + 80 Da) of eIF-5A (**a**) and that of the corresponding theoretically sulfated (**b**) and phosphorylated (**c**) peptides. The spectrum (**a**) was extracted from the full mass chromatogram (*m/z* 350–2000). The lower two theoretical spectra were calculated as the tryptic peptide plus SO_3_ (**b**) or HPO_3_ (**c**) based on the element composition. (**d**) The +3 charge state for MS/MS of the apparently sulfated peptide of residues 68–85 (*m/z* 728.52). (**e**) Western blot analysis of cytosolic and secreted re-eIF5A for tyrosine sulfation (upper panel) and eIF5A (lower panel) using YSP5-45-36. (**f**) Effects of hypoxia (60 min)/reoxygenation on the translocation of eIF5A to the *trans*-Golgi (labeled with syntaxin 6 [lower panel]) and on tyrosine sulfation (upper panel) or on the localization of eIF5A (middle panel) using YSP5-45-36.

To identify the type and amino acid site of this eIF5A modification, we analyzed purified secreted and cytosolic re-eIF5A via nanoLC-LTQ orbitrap MS. The molecular weight of the peptide corresponding to residues 68–85 was 80 Da higher than expected (Fig. 2a–c). Candidate modifications to explain this 80 Da increase included phosphorylation and sulfation. Figure 2a–c shows the +3 charge state mass spectra of the apparently (*m/z* = 728.65180, Fig. 2a) and theoretically (*m/z* = 728.65172, Fig. 2b) sulfated and the theoretically phosphorylated (*m/z* = 728.65489, Fig. 2c) tryptic peptides. Because the mass of (a) was much closer to (b) than to (c), we identified this modification as sulfation.

We also determined which residue within the peptide (68–85) was sulfated via ETD MS/MS using a LTQ-Orbitrap ETD mass spectrometer (Fig. 2d). Fragmenting the peptide via ETD MS/MS generated the sequence ions by cleaving the bonds along the backbone of the protonated peptide to yield the C and Z ion series, generating (NH_2_CR_1_HCONHR_2_CHCONH_2_ + H)^+^ for C_2_ and (H_2_ CR_3_CONHCR_4_H COOH + H)^+^ for Z_2_. We confirmed the C_3_, C_4_, C_5_, C_10_, C_11_, C_12_, C_13_ and C_15_ ions and Z_3_, Z_9_ and Z_13_ ions. The M/Z 1438.9 of Z_13_ and the *m/z* 1455.27 of Y_13_ indicated that the peptide sequence from 72 to 85 was not modified. However, the *m/z* 517.2 of C_3_ was equal to KYE plus 80, so we concluded that the tyrosine residue 69 was sulfated (Fig. 2d). Table 1 shows the ratio of the ion intensity of the sulfated peptide of the cytosolic form to that of the secreted form of re-eIF5A. The N-terminal peptide (27–34), which was not modified, was used as a control for comparison with the sulfated peptide. The ratio of the +2 charge state ion of the secreted to the cytosolic peptide corresponding to residues 27–34 was 0.38, and that of the peptide corresponding to residues 28–34 was 0.45. In contrast, the ratios of the +2 and +3 charge state ions of the sulfated peptide corresponding to residues 68–85 were 0.87 and 0.92, respectively. The ratios of the +2 and +3 charge state ions of the peptide corresponding to residues 69–85 were 0.82 and 1.91, respectively. These differences indicated that secreted re-eIF5A contains much more sulfated eIF5A than cytosolic re-eIF5A (Table 1).

We confirmed this finding by performing Western blot analysis of cytosolic and secreted re-eIF5A for sulfo-tyrosine and eIF5A (Fig. 2e). The ratio of sulfo-tyrosine expression in secreted/cytosolic re-eIF5A was 3.94 ± 1.02 (mean ± s.d.) (n = 4). Tyrosine sulfation, which lowers the pI value, is a common post-translational modification of secreted and plasma membrane proteins that plays a crucial role in the interactions of these proteins with their binding partners^13^,^14^. Tyrosine sulfation of these proteins is catalyzed by tyrosylprotein sulfotransferases (TPSTs), which are localized to the Golgi apparatus^15^, and this modification occurs in the *trans*-Golgi as the final modification before secretion^16^. Tyrosine sulfation of secreted eIF5A strongly supports the acquisition of its physiological function as an extracellular ligand.

### EIF5A rapidly translocates to the Golgi apparatus and is tyrosine sulfated in the *trans*-Golgi for secretion

In general, intracellular proteins that are ultimately secreted into the extracellular space are thought to require an N-terminal signal peptide (classical secretory pathway). However, evidence has accumulated that some proteins, including cytokines and growth factors, lacking an N-terminal signal peptide can be secreted (non-classical secretory pathway)^17^,^18^. Most tyrosine-sulfated proteins identified to date are secreted. These secretory proteins are tyrosine-sulfated in the *trans*-Golgi and are then sorted into membrane vesicles, which transport these proteins to the plasma membrane for secretion^14^,^16^. To investigate whether eIF5A actually translocates to the *trans*-Golgi for tyrosine sulfation upon stimulation, we collected the Golgi-enriched fraction (according to the method of Graham J.M.^19^) and examined the expression of eIF5A and its tyrosine sulfation level in response to hypoxia/reoxygenation. We confirmed the rapid accumulation and the marked tyrosine sulfation of eIF5A in the *trans*-Golgi (using syntaxin 6 as a specific marker^20^); these events peaked at 2 min after reoxygenation (Fig. 2f).

Next, we analyzed the subcellular localization of eIF5A in cultured cardiac myocytes and found that hypoxia/reoxygenation induced the translocation of eIF5A from the perinuclear region to the peripheral cytoplasm, such as within granules (Supplementary Fig. 4a; upper panels, indicated by arrows). Immunoelectron microscopy revealed that eIF5A was localized to granules that were immediately adjacent to or on the plasma membrane or to granules that were far from the plasma membrane, as if they were being secreted (Supplementary Fig. 4b; left, middle, and right panels, respectively, indicated by arrows). We also immunostained for eIF5A in ventricular tissues *in vivo*. In sham-operated rats and rats subjected to ischemia (30 min) alone, hardly any eIF5A expression in the cardiac myocytes was detected (Supplementary Fig. 5; upper left and right panels, respectively). In contrast, myocardial ischemia (30 min)/reperfusion (15 and 30 min) clearly increased eIF5A expression on the plasma membrane of many cardiac myocytes (Supplementary Fig. 5; lower panels).

### Secreted eIF5A induces apoptosis

**Figure 3:**
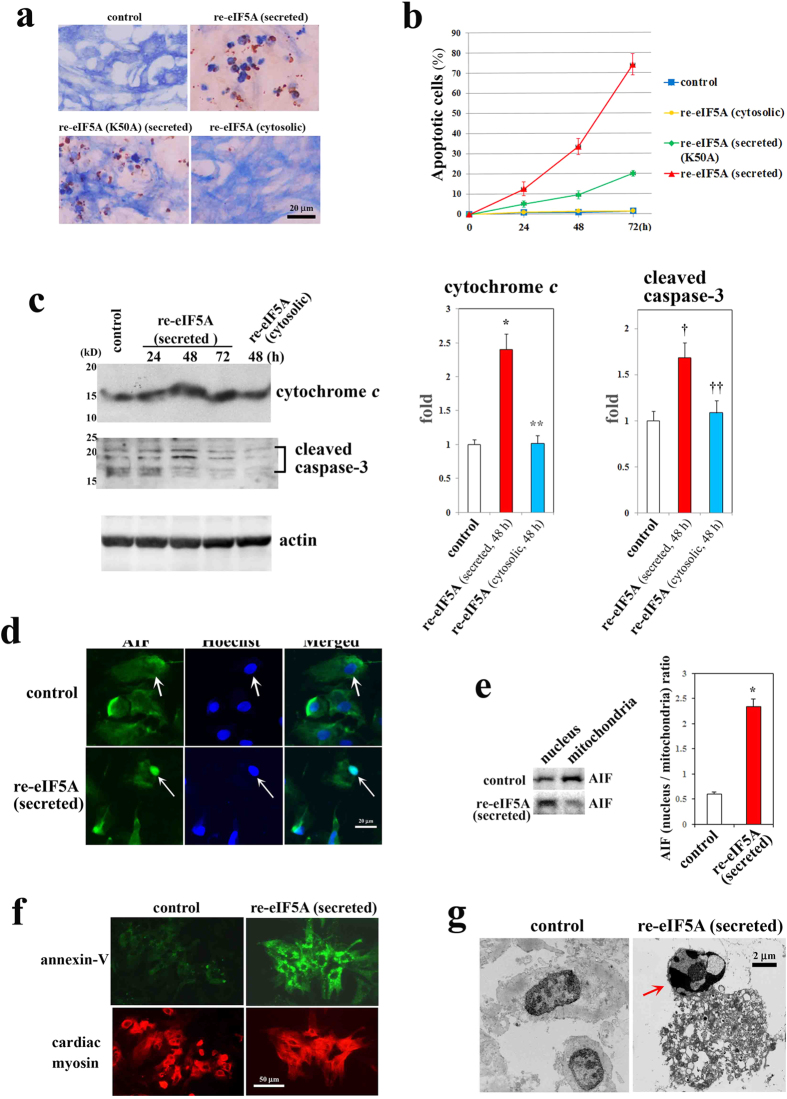
Induction of apoptosis in cardiac myocytes by eIF5A. (**a**–**f**) Effects of re-eIF5A protein (10 μg/ml) on cultured cardiac myocytes. (**a**) Induction of apoptosis in cardiac myocytes as determined by TUNEL staining (brown) and cardiac myosin immunostaining (blue). Representative images at 72 h after the addition of re-eIF5A protein. (**b**) A time course of the percentage of apoptotic cardiac myocytes, as determined by TUNEL staining, induced by re-eIF5A (cytosolic), re-eIF5A (secreted), or mutant re-eIF5A (K50A) (secreted). The data are expressed as the mean ± s.e.m. (n = 6 for each). (**c**) Western blot analysis of the effects of secreted re-eIF5A on cytochrome *c* release from the mitochondria (upper panel) and on the activation of caspase-3 (middle panel). A Western blot for actin was used as a loading control. *P = 0.0001 vs. control; **P = 0.0001 vs. re-eIF5A (secreted); ^†^*P*  = 0.0054 vs. control; ^††^*P* = 0.0107 vs. re-eIF5A (secreted) (mean ± s.e.m., n = 4 for each) (Dunnett's multiple comparison test). (**d**) Representative confocal images of the effect of secreted re-eIF5A on the subcellular localization of AIF. Nuclei (arrows) were stained with 1 μg/ml Hoechst 33342. (**e**) Western blot analysis of the effects of secreted re-eIF5A on subcellular translocation of AIF from the mitochondria to the nucleus. The ratio of the expression levels of AIF in the nuleus to the mitochondria were shown in the right panel. *P = 0.0001 vs. control (mean ± s.e.m., n = 4, unpaired *t*-test). (**f**) The induction of apoptosis in cardiac myocytes as determined by double-immunostaining for Annexin-V (upper panels, labeled with FITC) and cardiac myosin (lower panels, labeled with TRITC). (**g**) Electron microscopic determination of the hypercondensation of nuclear chromatin (an arrow) induced by secreted re-eIF5A. (**d**–**g**) Representative images at 48 h after the addition of secreted re-eIF5A.

Re-eIF5A from RCP (that is, secreted re-eIF5A, 10 μg/ml) potently induced the apoptosis of cultured cardiac myocytes, as shown by terminal deoxynucleotidyl transferase nick-end labeling (TUNEL) staining (brown color) and cardiac myosin immunostaining (blue color) (Fig. 3a). However, hardly any cardiac myocytes underwent apoptosis in the untreated control and cytosolic re-eIF5A (10 μg/ml)-treated groups (Fig. 3a). Secreted re-eIF5A mutated at a single amino acid (K50→A50) [eIF5A (K50A)] (10 μg/ml), which renders it unable to be hypusinated^12^, only partially induced apoptosis (Fig. 3a). This suggested that hypusination is important but not essential for the apoptosis-inducing function of secreted-eIF5A. We also found that hypusination appears not to affect significantly the secretion of eIF5A (data not shown). A time course depicting the percentage of apoptotic cardiac myocytes is shown in Fig. 3b. Secreted re-eIF5A significantly increased the cytosolic expression of cytochrome *c* and the cleaved form of caspase-3, both of which peaked at 48 h (Fig. 3c), and significantly induced the translocation of apoptosis-inducing factor (AIF) from the cytosol (mitochondria) to the nucleus in cardiac myocytes at 48 h as determined by Hoechst 33342 (1 μg/ml) staining and AIF immunostaining as well as Western blot for AIF (Fig. 3d,e). The induction of the apoptosis of cardiac myocytes by secreted re-eIF5A was further confirmed by Annexin-V staining (Fig. 3f) and by the hypercondensation of nuclear chromatin, as assessed by electron microscopy (Fig. 3g). Secreted re-eIF5A induced the phosphorylation of the mitogen-activated protein kinase (MAPK) family members, IκB and ATF2 (Supplementary Fig. 6), markedly activating ERK1/2 and moderately activating other MAPK members, Akt, and signal transducers and activators of transcription (STATs) (Supplementary Fig. 7a,b). Whereas cytosolic-re-eIF5A did not activate these signaling pathways (data not shown).

This activation confirmed that ERK activity is the most sensitive marker of the apoptosis-inducing humoral factor examined in the present study. Ataxia-telangiectasia mutated (ATM) is a serine/threonine kinase that signals to arrest the cell cycle or induce apoptosis by phosphorylating its downstream target p53 in response to DNA damage caused by oxidative stress. Secreted re-eIF5A increased the phosphorylation of ATM at Ser1981 and of p53 at Ser15 and Ser20, but not Ser46; these events peaked at 8–16 h after stimulation (Supplementary Fig. 8a). A specific inhibitor of p53 (pifithrin-α) significantly promoted apoptosis (Supplementary Fig. 8a,c), indicating that the ATM-p53 pathway plays a protective role against apoptosis induced by secreted re-eIF5A (10 μg/ml). The apoptosis of mammalian cells occurs via caspase-dependent and caspase-independent pathways. The activation of poly(ADP-ribose) polymerase-1 (PARP-1) signals the mitochondria to release AIF, which mediates the apoptotic signal in a caspase-independent manner^21^. We found that a PARP-1 inhibitor (3-aminobenzamide) and a caspase inhibitor (Z-VAD-fmk) significantly suppressed secreted re-eIF5A-induced apoptosis by approximately 30% and 70%, respectively, indicating that both pathways contribute to this apoptotic activity (Supplementary Fig. 9a,b).

### How does secreted eIF5A activate apoptotic signaling?

**Figure 4:**
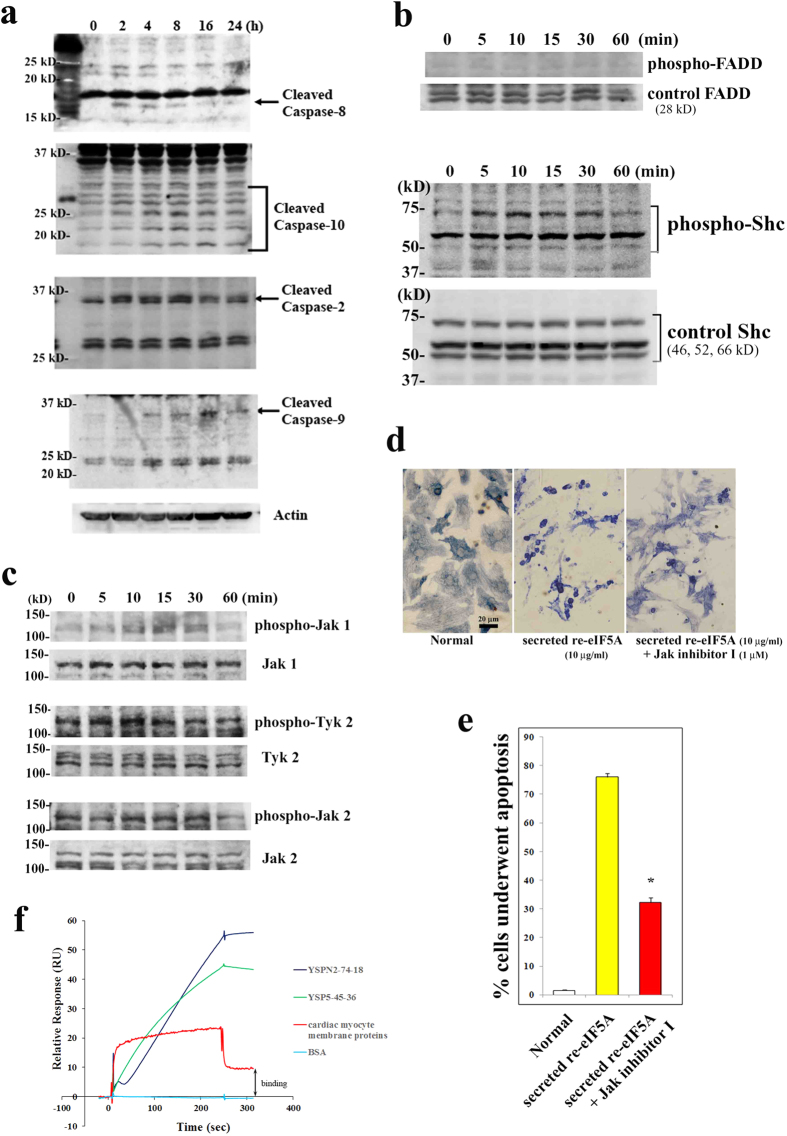
Upstream signaling mechanisms induced by secreted eIF5A in cardiac myocytes. (**a**) Secreted re-eIF5A (10 μg/ml) activated initiator caspases, including caspase-8 (IMG-5703; IMGENEX), caspase-10 (ab25045; Abcam), caspase-2 (ab2251; Abcam), and caspase-9 (#9508; Cell Signaling), in cultured cardiac myocytes. The anti-actin antibody was sc-1616 from Santa Cruz. (**b**) Secreted re-eIF5A (10 μg/ml) rapidly increased the phosphorylation of Shc at Tyr 239/240 (#2434; Cell Signaling) (lower panels), but not FADD at Ser 191 (sc-33399; Santa Cruz) (upper panels), in cultured cardiac myocytes. The control antibodies were anti-Shc (sc-967; Santa Cruz) and anti-FADD (ab24533; Abcam). (**c**) Secreted re-eIF5A (10 μg/ml) increased the phosphorylation of Jak 1 at Tyr 1022/1023 (sc-16773; Santa Cruz) and Tyk 2 at Tyr 1054/1055 (sc-11763), but not Jak 2 at Tyr 1007/1008 (sc-21870). The control antibodies were anti-Jak 1 (sc-295), anti-Tyk 2 (sc-169), and anti-Jak 2 (sc-34479). (**d**,**e**) The effects of Jak inhibitor I (Santa Cruz; 1 μM, pretreated for 24 h) on the induction of apoptosis in cardiac myocytes by secreted re-eIF5A (10 μg/ml, 72 h) as determined by TUNEL staining (brown) and cardiac myosin immunostaining (blue), as shown in Fig. 3a. *P < 0.0001 vs. secreted re-eIF5A alone (mean ± s.e.m., n = 4 each) (Dunnett's multiple comparison test) (**e**). (**f**) SPR analysis of the interaction between secreted re-eIF5A and cardiac myocyte membrane proteins. The surface of the sensor chip was coupled with 10μg/ml of secreted re-eIF5A in 10 mM acetate buffer (pH 5.5) at a flow rate of 5 μl/min. Then, cardiac myocyte membrane proteins (100 μg/ml in HBS-EP buffer) were analyzed for their interaction with the surface at a flow rate of 10 μl/min for 4 min. The anti-eIF5A mAbs (YSP5-45-36 and YSPN2-74-18, 1 μg/ml for each) and BSA (100 μg/ml) were used as positive and negative controls, respectively. All experiments were performed at least in triplicate. The results shown are from one representative experiment.

Next, we analyzed upstream apoptotic signaling pathways, including initiator caspases such as caspase-8, which is recruited and activated by a death domain-containing adaptor protein such as Fas-associated death domain (FADD) upon DR binding. We confirmed that secreted re-eIF5A significantly activated caspases −8 (2.59 ± 0.21 [mean ± s.e.m.] fold at 2 h, n = 3 for each, *P* = 0.0174 vs. control [0 h] [paired *t*-test]), −10 (2.26 ± 0.11 fold at 8 h, *P* = 0.0073), −2 (2.43 ± 0.18 fold at 8 h, *P* = 0.0160), and −9 (3.52 ± 0.45 fold at 16 h, *P* = 0.0306) in cultured cardiac myocytes within 2–4 h after stimulation (Fig. 4a). We further examined whether secreted re-eIF5A activates FADD, which mediates DR signaling, or Shc, which mediates receptor tyrosine kinase signaling and activates the MAPK pathway. We found that secreted re-eIF5A rapidly activated Shc (2.77 ± 0.07 [mean ± s.e.m.] fold at 10 min, n = 3 for each, *P* = 0.0014 vs. control [0 min] [paired *t*-test]) but not FADD (0.93 ± 0.06 fold at 10 min, *P* = 0.3576) (Fig. 4b). This result suggested that secreted re-eIF5A induces apoptotic signaling via one of the various types of receptors, including growth factor receptors, cytokine receptors, and G protein-coupled receptors, rather than via a known DR, such as TNF-R1, Fas, DR3, DR4, or DR5.

Janus family tyrosine kinases (Jaks) function immediately downstream of various cytokine receptors and play a critical role in cytokine signaling by recruiting and phosphorylating STATs, which subsequently dimerize and translocate to the nucleus. Because the phospho-kinase array showed that STATs were activated by secreted re-eIF5A (Supplementary Fig. 7a,b), we investigated the roles of the Jak/STAT pathway in secreted re-eIF5A-induced apoptotic signaling. Secreted re-eIF5A significantly activated Jak1 (2.22 ± 0.01 [mean ± s.e.m.] fold at 15 min, n = 3 for each, *P* = 0.0001 vs. control [0 min] [paired *t*-test]) and Tyk 2 (2.03 ± 0.19 fold at 10 min, *P* = 0.0337), but not Jak 2 (1.11 ± 0.12 fold at 15 min, *P* = 0.4845) (Fig. 4c). Jak inhibitor I (Santa Cruz Biotechnology; 1 μM, pretreated for 24 h) significantly reduced the induction of apoptosis in cardiac myocytes (Fig. 4d,e). This result indicated that the Jak/STAT pathway at least partially mediates secreted eIF5A-induced apoptotic signaling and suggested that secreted eIF5A binds to a cell surface receptor with cytokine receptor-like structure. To investigate whether secreted eIF5A actually binds to cell surface molecules, we performed surface plasmon resonance (SPR) to analyze the interaction between secreted re-eIF5A and cell membrane proteins that were extracted from cardiac myocytes subjected to hypoxia (60 min)/reoxygenation (30 min). We found significant binding between secreted re-eIF5A and cell membrane proteins, suggesting that a cell surface receptor interacts with secreted eIF5A. Strong binding was observed between secreted re-eIF5A and the anti-eIF5A mAbs (YSP5-45-36 and YSPN2-74-18), showing the specificity of these mAbs. And, non-specific binding with BSA was not observed (Fig. 4f).

### Anti-eIF5A suppresses hypoxia/reoxygenation-induced apoptosis and ischemia/reperfusion injury

Next, we developed anti-eIF5A neutralizing mAbs against a region containing the hypusination and tyrosine sulfation sites and an N-terminal region and analyzed the effects of these neutralizing mAbs on hypoxia/reoxygenation-induced signaling and apoptosis. Treatment with both anti-eIF5A neutralizing mAbs (YSP5-45-36 and YSPN2-74-18) significantly suppressed the hypoxia (60 min)/reoxygenation (15 min)-induced activation of ERK1/2 in cardiac myocytes (Supplementary Fig. 10) and the hypoxia (11 h)/reoxygenation (48 h)-induced cytosolic release of cytochrome *c* and activation of caspase-3 (Supplementary Fig. 11a,b).

Together, these changes resulted in a significant suppression of apoptosis induced by hypoxia (15 h)/reoxygenation (72 h) (Supplementary Fig. 12a,b). This result indicated that hypoxia/reoxygenation-induced apoptotic signaling is predominantly mediated by secreted eIF5A. The neutralizing activity of YSP5-45-36 appeared to be greater than that of YSPN2-74-18, suggesting that this region, which includes the hypusination and tyrosine-sulfation sites, may play a critical role in the binding of secreted eIF5A to its receptor. YSP5-45-36 also significantly suppressed UV irradiation-induced signaling and apoptosis in cardiac myocytes (Supplementary Fig. 13, Supplementary Fig. 14a,b, and Supplementary Fig. 15a,b). This finding suggested that a mechanism of apoptosis involving the autocrine secretion of eIF5A may be common to various types of oxidative stress.

**Figure 5:**
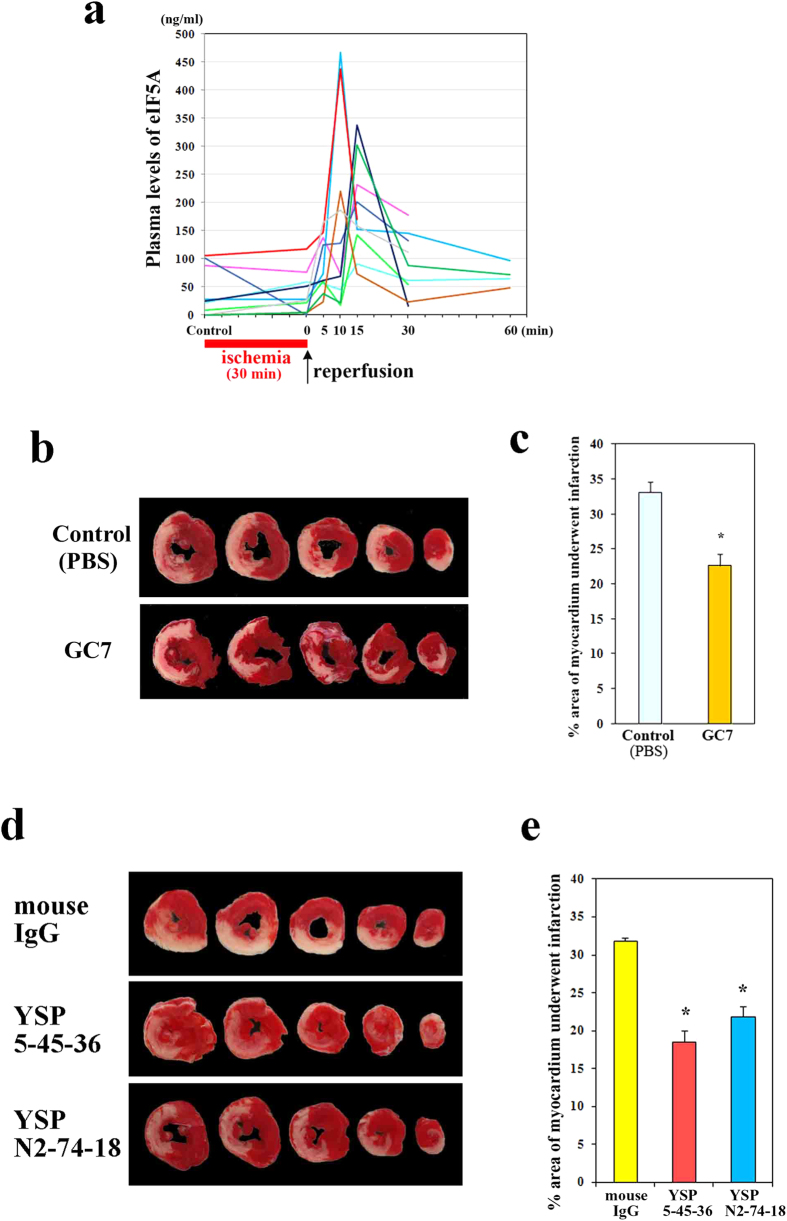
Myocardial ischemia/reperfusion induces the release of eIF5A into the circulation, and the neutralization of eIF5A suppresses myocardial ischemia/reperfusion injury *in vivo*. (**a**) The plasma levels of eIF5A in rats that were subjected to myocardial ischemia/reperfusion *in vivo*. *P* values were calculated using a paired *t*-test (n = 10). (**b**) Pretreatment with GC7 (Biosearch Technologies, Inc., 1 mg/kg intraperitoneally, daily from 5 days before until 1 day before ischemia/reperfusion) reduced myocardial ischemia/reperfusion injury. Representative cross-sections of a heart stained with triphenyl tetrazolium chloride (TTC) from the control group (upper panel) and the GC7-treated group (lower panel). (**c**) The infarct size of the GC7-treated rats was significantly smaller than that of the control PBS-treated rats (n = 12, *P = 0.001) (Mann-Whitney *U*-test; *P* values corrected using the Bonferroni method). (**d**) The anti-eIF5A neutralizing mAbs (YSP5-45-36 and YSPN2-74-18) reduced myocardial ischemia/reperfusion injury. Representative cross-sections of a heart from the mouse IgG-treated group (upper panel), the anti-eIF5A mAb (YSP5-45-36)-treated group (middle panel), and the anti-eIF5A mAb (YSPN2-74-18)-treated group (lower panel). (**e**) The infarct size in the anti-eIF5A mAb (YSP5-45-36 and YSPN2-74-18)-treated groups was significantly smaller than that in the mouse IgG-treated group (n = 12, *P < 0.0001 for both) (Dunnett's multiple comparison test).

We developed a sandwich enzyme-linked immunosorbent assay (ELISA) using YSPN2-74-18 and YSP5-45-36 (Supplementary Fig. 16) and measured the plasma levels of eIF5A in rats that were subjected to myocardial ischemia/reperfusion. No significant changes in the plasma eIF5A levels were observed between the control condition (before ischemia) and after 30 min of ischemia (immediately before reperfusion). However, these levels began to increase after reperfusion and were significantly increased 10–15 min after reperfusion (262.1 ± 39.0 ng/ml [mean ± s.e.m.]) compared with immediately before reperfusion (38.6 ± 11.8 ng/ml; n = 10, *P* = 0.0001) (Fig. 5a). The plasma levels of eIF5A were significantly increased in rats whose hearts were subjected to UV irradiation *in vivo* (Supplementary Fig. 17).

Next, we analyzed the effects of N1-guanyl-1,7-diaminoheptane (GC7), a hypusination inhibitor that blocks deoxyhypusine synthase (DHS) function, and the anti-eIF5A neutralizing mAbs in a rat model of myocardial ischemia/reperfusion. The mean (±s.e.m.) infarct size (expressed as a percentage of the size of the left ventricle) in the GC7-treated group was significantly smaller (22.61 ± 1.60%, n = 12) than that in the control PBS-treated group (33.09 ± 1.45%, n = 12) (*P* = 0.001, Fig. 5b,c), indicating the critical, but not essential, role of hypusination in ischemia/reperfusion injury as in an *in vitro* model. Treatment with either anti-eIF5A neutralizing mAb (5 mg/kg intravenously, 10 min before reperfusion) significantly reduced the infarct size (YSP5-45-36, 18.62 ± 1.46%; YSPN2-74-18, 21.93 ± 1.23%; n = 12) compared with the control IgG treatment (31.87 ± 0.42%, n = 12, *P* < 0.0001 for both). (Fig. 5d,e). The effects of these mAbs *in vivo* were consistent with their effects *in vitro*. For YSP5-45-36, doses higher than 3 mg/kg (up to 10 mg/kg) did not exert significantly enhanced effects. For YSPN2-74-18, no significant dose response was observed from 1 mg/kg to 10 mg/kg (Supplementary Fig. 18a,b).

## Discussion

The molecular mechanism underlying the cellular response to oxidative stress is only partially understood. The activation of Src, Ras and Raf-1 has been reported in response to UV exposure^22^ and hypoxia/reoxygenation^7^,^23^. PARP-1 mediates caspase-independent apoptotic signaling and contributes to reperfusion injury^24^. We showed that PARP-1 partially contributes to secreted eIF5A-induced apoptotic signaling. Mechanical stretching of cardiac myocytes causes the release of angiotensin II, which, in turn, activates signaling pathways that lead to cell hypertrophy^25^. This response represents an exquisite autocrine mechanism in which cardiac myocytes respond to mechanical stress by increasing the expression of contractile proteins via the release of angiotensin II. In contrast, the present study showed that cardiac myocytes fail to adapt to excessive oxidative stress, resulting in apoptosis in response to secreted eIF5A. There may be a balance between pro-apoptotic and anti-apoptotic factors in the cellular response to oxidative stress, and this balance may be lost if excessive oxidative stress induces the cardiac myocytes to secrete sufficient levels of eIF5A to induce apoptosis.

Although eIF5A was identified as an eIF^26^, its actual function remains unclear. eIF5A performs multiple intracellular functions and is involved in cell growth (such as translation initiation, translation elongation^27^, and the cell cycle^28^) and cell death (apoptosis). The translation initiation activity of eIF5A correlates with its hypusination^29^,^30^, which is also critical for cell cycle progression^28^. Because eIF5A is not essential for general protein synthesis, eIF5A may be required for the translation of certain mRNAs or may be involved in other processes of cell metabolism^31^,^32^. In the present study, we demonstrated significant binding between secreted re-eIF5A and cardiac myocyte membrane proteins (Fig. 4f), suggesting a possible ligand-receptor interaction. We also showed that the upstream apoptotic signaling events immediately downstream of the receptor may at least partially involve the Jak/STAT pathway. The DR TWEAK signals via the Jak/STAT pathway to induce apoptosis^33^, although the receptor for secreted eIF5A does not appear to be a known DR. Thus, our findings revealed a novel extracellular function of a post-translationally modified form of eIF5A as a pro-apoptotic ligand.

**Figure 6:**
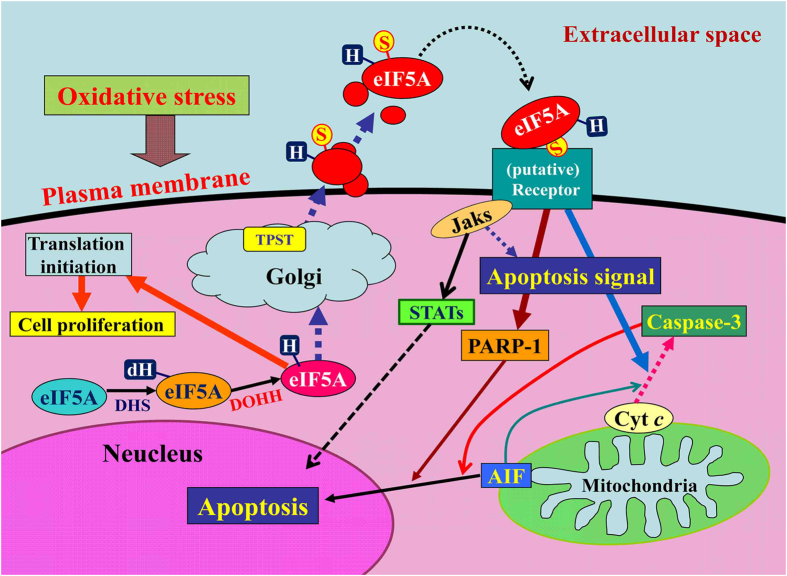
A model for the mechanism by which oxidative stress induces apoptosis via the autocrine secretion of eIF5A (proposed based on the data obtained in the present study). AIF, apoptosis-inducing factor; cyt *c*, cytochrome *c*; dH, deoxyhypusine; DHS, deoxyhypusine synthase; DOHH, deoxyhypusine hydroxylase; H, hypusine; Jaks, Janus kinases; S, sulfated; PARP-1, poly (ADP-ribose) polymerase-1; STATs, signal transducers and activators of transcriptions; TPST, tyrosylprotein sulfotransferase.

Hypusination may play an important role in receptor binding or signaling (Fig. 6). This hypothesis is supported by the known structural characteristics of eIF5A^34^. We posit that the extracellular function of eIF5A as a pro-apoptotic ligand may be the major function of this unique protein. Because eIF5A is ubiquitously and abundantly expressed in various cell types, this autocrine mechanism may mediate a common cellular response to oxidative stress. Here, we propose referring to this tyrosine-sulfated and hypusinated form of secreted eIF5A as Oxidative stress-Responsive Apoptosis Inducing Protein (ORAIP). The present study provides a novel specific biomarker and a potential therapeutic target for oxidative stress-induced cell injury.

## Methods

### Cells and collection of the hypoxia/reoxygenation-conditioned PBS

Primary ventricular cardiac myocytes were prepared from neonatal rats as previously described^7^. The cells were cultured for two days until confluence. Hypoxic conditions (95% N2, 5% CO2, and less than 0.1% O2) were generated as described previously^7^. After incubating the cells under hypoxic conditions for 60 min, the cells were reoxygenated by immediately replacing the hypoxic PBS with normoxic PBS for 10 min. We collected the supernatant PBS after 10 min of reoxygenation; this sample was referred to as RCP. We also collected the supernatant PBS from non-stimulated cardiac myocytes incubated for 10 min under normoxia; this sample was referred to as CCP. We concentrated proteins with a molecular weight >10 kD in the RCP and CCP, and collected fractions (>10 kD) using centripreps (YM-10; Millipore Corporation). These samples were separated via chromatofocusing, and fractions inducing high ERK activity in cardiac myocytes were subjected to 2-D gel electrophoresis.

### Chromatofocusing

Concentrated RCP (595 μg of total protein collected from 3.9 × 10^8^ cells) and CCP (698 μg of total protein collected from 10.0 × 10^8^ cells) were loaded on a MONO-P (5 mm × 200 mm) column (GE Healthcare) connected to a GILSON HPLC system at a flow rate of 1 ml/min. The samples were equilibrated with Solution A (0.025 M Bis-Tris, pH 7.1) and eluted with Solution B (10% Poly Buffer74, pH 4.0), and then, the column-bound components were washed out with Solution C (1 M NaCl/10% Poly Buffer74, pH 4.0).

### Monitoring ERK activity induced by the chromatofocusing fractions

Cultured cardiac myocytes were treated with aliquots of each fraction after chromatofocusing RCP or CCP (188 ml collected from 2.35 × 10^8^ cells in each group) for 5 min. Then, the culture media were aspirated immediately, and the cells were frozen in liquid nitrogen. The procedures for Western blot analysis using rabbit polyclonal phospho-specific anti-ERK1/2 (Thr202/Tyr204) and total anti-ERK1/2 antibodies (Cell Signaling, Inc.) were performed as previously described^8^.

### 2-D gel electrophoresis

Aliquots of the combined fractions (49–52) of RCP (14.6 μg) or CCP (6.0 μg) consisting of 264 ml collected from 3.3 × 10^8^ cells in each group were subjected to 2-D gel electrophoresis.

### Cloning and construction of plasmids

Human eIF5A cDNA was amplified via RT-PCR using total RNA isolated from SaSO2 cells (a human osteoblast-like cell line) and was subcloned into the *Eco*RI/*Xho*I site of the pcDNA4/Myc-His vector (Invitrogen). Then, a FLAG- and His-tagged eIF5A construct was generated via PCR using the following primers: forward, 5′-CACCGAATTCAAAATGGCAGATGACT-3′; reverse, 5′-ATATACTCGAGTCAGTGATGGTGATGGTGGTGCTTGTCATCGTCGTCCTTGTAATCTTTTGCCATGGCCTTGATTG-3′. This construct was then subcloned into the pcDNA3.1 Directional TOPO vector (Invitrogen). Using this plasmid DNA as a template, a vector expressing human eIF5A mutated at a single amino acid (K50 → A50) [eIF5A (K50A)], which cannot be hypusinated, was constructed using the QuickChange II Site-Directed Mutagenesis Kit (Stratagene) used according to the manufacturer’s instructions.

### Recombinant eIF5A proteins

FLAG- and His-tagged eIF5A, Myc- and His-tagged eIF5A, and mutant eIF5A (K50A) were transfected into a quail muscle cell line (CRL-1962, American Type Culture Collection) using FuGENE HD Transfection Reagent (Roche). Forty-eight hours after transfection, the cells were subjected to hypoxia for 20 min followed by reoxygenation for 10 min. The RCP was collected and concentrated; then, the recombinant proteins were purified using the Ni-NTA Purification System (Invitrogen). The proteins were further purified via two-step gel filtration chromatography using Superdex 200^TM^ 10/300 GL and Superdex 75^TM^ 10/300 GL columns (GE Healthcare). Cytosolic re-eIF5A was also collected from the transfected cells under native conditions. The purity of re-eIF5A was more than 95% as determined by silver staining and Western blot (data not shown).

### Western blot analysis of Myc-and FLAG-tagged re-eIF5A proteins

Myc- and His-tagged re-eIF5A protein was collected from the cytosolic fraction of quail muscle cells transfected with the eIF5A-myc-His vector, and FLAG- and His-tagged re-eIF5A protein was purified from the RCP using the Ni-NTA Purification System (Invitrogen) followed by gel filtration under native conditions. These proteins were mixed and subjected to 2-D gel electrophoresis followed by transfer to a PVDF membrane. The membrane was first analyzed via Western blot using an anti-Myc mAb (Invitrogen), a biotinylated anti-mouse IgG, and the Vectastain ABC-AP Kit (Vector), followed by development via chemiluminescence using alkaline phosphatase. Then, the membrane was probed with an anti-FLAG mAb (M2, Sigma) and a horseradish peroxidase (HRP)-labeled anti-mouse IgG (Cell Signaling), followed by development using the Konica Immunostain HRP-1000 Kit (Konica, Tokyo, Japan). We did not observe a significant difference in the pI value between the Myc- and His-tagged cytosolic re-eIF5A protein and the FLAG- and His-tagged cytosolic re-eIF5A protein based on 2-D gel electrophoresis followed by Western blot analysis, indicating that these two tags did not cause significant shift in pI (data not shown).

### Western blots for cytochrome *c* release from mitochondria and for caspase-3 activation

The anti-cytochrome *c* (H104) and anti-actin (I-19) polyclonal antibodies were obtained from Santa Cruz Biotechnology Inc., the anti-caspase-3 mAb (Asp175) (5A1E) was purchased from Cell Signaling Technology, and another anti-cytochrome *c* mAb (7H8.2C12) was obtained from Lab Vision Corp. The cytosolic fractions of the cells were prepared as supernatants of cell lysates centrifuged at 20,000 × *g* for 20 min as described elsewhere^35^. Western blot using an anti-actin antibody was performed as a loading control.

### Antibodies

An anti-sulfo-tyrosine mAb (Sulfo-1C-A2) was obtained from Millipore, and an anti-syntaxin 6 mAb (clone EP7665, ab140607) was purchased from Abcam.

### Anti-eIF5A antibodies

A rabbit anti-eIF5A polyclonal antibody (J-M) was generated against a human eIF5A peptide (residues 38–57) that included the hypusination site coupled to keyhole limpet hemocyanin (KLH). A mouse anti-eIF5A monoclonal antibody (mAb) (clone YSP5-45-36) was generated against a human eIF5A peptide (residues 44–72) that included the hypusination site and tyrosine 69, a sulfation site, coupled to KLH. Another mouse anti-eIF5A mAb (clone YSPN2-74-18) was generated against a human eIF5A peptide (residues 7–33) near the N-terminal region that was coupled to KLH.

### Western blot analysis of cultured cardiac myocytes

The procedures for the Western blot analyses were the same as those described elsewhere^9^.

### Immunofluorescence

Immunofluorescence staining of cultured cardiac myocytes and myocardial tissues was performed using Tyramide Signal Amplification (TSA) technology for fluorescence (TSA^TM^ Biotin System, PerkinElmer). Double-immunostaining for cardiac myosin was performed as described elsewhere^9^. To stain for AIF, the cells were fixed with 4% paraformaldehyde in PBS for 15 min and then incubated in the rabbit anti-AIF mAb (E20; Epitomics Inc.). Confocal microscopy was performed using an LSM510 laser scanning microscope (Zeiss). To stain for Annexin-V, the cells were incubated in biotinylated Annexin-V in 1X binding buffer (Annexin V-Biotin Apoptosis Detection Kit, BioVision Inc.) for 5 min and then fixed with 2% paraformaldehyde in PBS for 15 min.

### Subcellular fractionation

Mitochondrial and nuclear fractions were separated from cultured cardiac myocytes using Cell Fractionation Kit-Standard (ab109719, Abcam). Then, the fractions were subjected to Western blot using an anti-AIF mAb.

### Electron microscopy

The cells were fixed in 2% glutaraldehyde, post-fixed in 2% osmium tetroxide, and embedded in resin. Ultrathin sections were prepared, stained with uranyl acetate and lead citrate, and examined under an electron microscope (H-7000, HITACHI, Japan). For immunoelectron microscopy, cultured cardiac myocytes were subjected to hypoxia for 60 min followed by reoxygenation for 10 min, fixed in 4% paraformaldehyde for 2 h, washed with PBS and dehydrated using a cold graded ethanol series (50–100%). The myocytes were embedded in LR White resin (Nisshin EM, Co. Ltd., Japan):100% ethanol (1:1) for 2 h and then embedded in pure LR White resin, which was polymerized under ultraviolet light irradiation at 4 °C overnight. Ultrathin sections were prepared, blocked with 1% bovine serum albumin in PBS and incubated in an anti-eIF5A antibody (J-M) overnight. After washing with PBS, the sections were incubated in Protein A-conjugated gold colloidal particles (20 nm) (EY Laboratories, Inc.).

### TUNEL staining and cardiac myosin immunostaining

We used the *In Situ* Apoptosis Detection Kit (TAKARA BIO Inc., Japan) followed by diaminobenzidine (DAB) reaction (brown color) for TUNEL staining. Additionally, we used an anti-cardiac myosin mAb (clone CMA19^37^) followed by alkaline phosphatase-labeled anti-mouse IgG (Santa Cruz Biotechnology); the samples were then reacted with an alkaline phosphatase substrate (alkaline phosphatase substrate kit III, Vector Laboratories) to produce a blue reaction product for cardiac myosin staining.

### Mass spectrometry

re-eIF5A in 200 mM NH_4_HCO_3_ was reduced with 45 mM DTE, alkylated with acrylamide and then digested using trypsin^36^. The extracted ion chromatogram was obtained using a Thermo Fisher LTQ Orbitrap ETD connected to the Dina nanoLC apparatus. The analytical conditions for mass spectrometry were as follows: *ESI ionization:* capillary temp, 200 °C; spray, 2.5 kV; MS range, 350–2000; and MS resolution, 60000. The extracted ion chromatograms at the +2 and +3 charge states for the sulfated peptide of residues 68–85 of eIF5A and at the +2 charge state of the unmodified N-terminal peptide of residues 27–34. The mass tolerance window was ±3 ppm. Additionally, for MS/MS and ETD, the following conditions were used: Collision energy, 35%; collision gas, N_2_ for ETD; and ETD reaction time, 100 ms. For nanoLC, we used an HiQ Sil C18w-3 0.1 × 100 mm column. Solvent A was 0.1% HCOOH in H_2_O, and solvent B was 0.1% HCOOH in 90% CH_3_CN. The solvent program was solvent B; 0% for 30 min followed by a gradient from 0% to 70% for 45 min. The flow rate was 0.3 μl/min.

### Myocardial ischemia/reperfusion

Wistar rats (male, 7-weeks-old) were used in the *in vivo* study. The rats were euthanized 24 h after reperfusion, and the rats that died before 24 h of reperfusion were excluded from the analysis. The procedures for myocardial ischemia/reperfusion were performed as described elsewhere^38^. All animals were randomized to the control and treatment groups, and the investigators were not blinded to the treatments. All animal experiments were performed in accordance with the Guide of The Japanese Association of Laboratory Animal Facilities of National University Corporations and with the approval of the institutional animal care committee. Power estimates were calculated based on the detection of significant differences (95% power with an estimated effect magnitude of 18–45%).

### Direct UV irradiation of the rat hearts

Rats were anesthetized and ventilated using a respirator as performed for myocardial ischemia/reperfusion. After lateral thoracotomy, the heart was exposed to UV irradiation by opening the chest.

### SPR analysis

We performed SPR analysis using a BIAcore 2000 SPR biosensor (GE Healthcare). Secreted re-eIF5A was immobilized on a sensor chip CM5 using the amine coupling method. Plasma membrane proteins, which were extracted from cardiac myocytes that had been subjected to hypoxia (60 min)/reoxygenation (30 min) using a ProteoJET Membrane Protein Extraction Kit (Thermo Fisher Scientific Inc.), were analyzed for their interaction with secreted re-eIF5A protein. The anti-eIF5A mAbs (YSP5-45-36 and YSPN2-74-18) and bovine serum albumin (BSA) were used as positive and negative controls, respectively.

## Additional Information

**How to cite this article**: Seko, Y. *et al*. Secreted tyrosine sulfated-eIF5A mediates oxidative stress-induced apoptosis. *Sci. Rep*. **5**, 13737; doi: 10.1038/srep13737 (2015).

## Supplementary Material

Supplementary Information

## Figures and Tables

**Table 1:** Ratio of Ion intensity of the sulfated peptide from 68 to the 85 residue of eIF5A between the cytosolic and the secreted RCP fraction using the extracted ion chromatogram.

Peptide Residue	Molecular Weight	*m/z*	Charge State	Ion Intensity	(B) Cytosolic eIF5A	Ratio
				(A) Secreted eIF5A		(A)/(B)
**(1) None modified N-terminal peptide from the 27 to 34 residue**						
27–34 KNGFVVLK	904.6	452.78301	+2	1.26E + 05	3.28E + 05	0.38
28–34 NGFVVLK	776.5	388.73572	+2	1.93E + 06	4.28E + 06	0.45
**(2) Sulfated peptide from the 68 to 85 residue**						
68–85 M(O)&Y(+SO3)	2198.9	1100.46810	+2	1.99E + 05	3.06E + 05	0.65
68–85 M(O)&Y(+SO3)	2198.9	733.98116	+3	1.17E + 06	2.51E + 06	0.44
68–85 Y(+SO3)	2182.9	1092.47066	+2	5.26E + 05	6.06E + 05	0.87
68–85 Y(+SO3)	2182.9	728.64953	+3	3.55E + 06	3.84E + 06	0.92
69–85 M(O),Y(+SO3)	2070.8	1036.42080	+2	7.11E + 05	1.32E + 06	0.54
69–85 Y(+SO3)	2054.8	1028.42337	+2	2.22E + 06	2.71E + 06	0.82
69–85 Y(+SO3)	2054.8	685.95134	+3	1.41E + 05	7.36E + 04	1.91
