# Supplementary material for: Secreted tyrosine sulfated-eIF5A mediates oxidative stress-induced apoptosis
Source: Sci Rep. 2015 Sep 8;5:13737. doi: 10.1038/srep13737 (PMC4562266; doi:10.1038/srep13737)
Supplement: Supplementary Information [file srep13737-s1.pdf]

(Supplementary Information for)

Secreted tyrosine sulfated-eIF5A mediates oxidative stress-induced apoptosis

Yoshinori Seko,[1,3]* Tsutomu Fujimura,[2] Takako Yao[1], Hikari Taka,[2] Reiko Mineki,[2] Ko Okumura,[3] Kimie Murayama[2]

[1]*Dvision of Cardiovascular Medicine, The Institute for Adult Diseases, Asahi Life Foundation, 2-2-6 Nihonbashi-Bakurocho, Chuo-ku, Tokyo 103-0002, Japan*
[2]*Division of Proteomics and Biomolecular Science, BioMedical Research Center, Graduate School of Medicine, Juntendo University, Bunkyo-ku, Tokyo 113-8421, Japan*
[3]*Department of Immunology, Graduate School of Medicine, Juntendo University, Bunkyo-ku, Tokyo 113-8421, Japan*

*Corresponding author. Yoshinori Seko, M. D., Ph. D., Division of Cardiovascular Medicine, The Institute for Adult Diseases, Asahi Life Foundation, 2-2-6 Nihonbashi-Bakurocho, Chuo-ku, Tokyo 103-0002, Japan.
E-mail: sekoyosh-tky@umin.ac.jp FAX: +81-3-3639-5520
TEL: +81-3-3639-5501

Supplementary Figure 1: Reoxygenation-conditioned medium (RCM; 50 μg/ml) phosphorylates ERK1/2, SAPKs, p38MAPK in cultured rat cardiac myocytes. The procedures for Western analysis were as described previously[9]. The experiments were performed at least in triplicate. The results shown are from one representative experiment.

Supplementary Figure 2: Reoxygenation-conditioned media (RCM)-induced apoptosis of cultured rat cardiac myocytes as determined by TUNEL staining (brown) and cardiac myosin immunostaining (blue). Cardiac myocytes at 72 h after addition of RCM (total protein concentrations; **b**, 25 μg/ml; **c**, 50 μg/ml; **d**, 100 μg/ml, respectively) and without RCM (**a**). Higher magnification revealed granule-like brown TUNEL-stainings (arrows) in almost all round-shaped cardiac myocytes (**e**). RCM induced apoptosis of cardiac myocytes dose-dependently (**f**). At least 800 cells were counted in each group (mean ± s.e.m., n=4 for each)

Supplementary Figure 3: Western analysis of Myc-tagged-cytosolic and FLAG-tagged-secreted recombinant eIF5A proteins. Myc- and His-tagged cytosolic-re-eIF5A protein and FLAG- and His-tagged secreted-re-eIF5A protein were mixed and subjected to a 2-D gel electrophoresis, and analyzed by Western blot, first with anti-Myc mAb developed by chemiluminescence using alkalinephosphatase, and then, with anti-FLAG mAb developed by immunostaining using horseradish peroxidase (HRP). Arrows indicate A and A', unhypusinated; B and B', deoxyhypusinated; C and C', hypusinated form, respectively.

Supplementary Figure 4: Expression of eIF5A in cardiac myocytes subjected to hypoxia/reoxygenation *in vitro*. (a) Immunofluorescent localization of eIF5A protein in cultured cardiac myocytes in response to hypoxia/reoxygenation as determined by double-immunostaining with an anti-eIF5A antibody (J-M, upper panels) and an anti-cardiac myosin (CMA19, lower panels)[21]. **(b)** Immunoelectron microscopic subcellular localization of eIF5A protein in cultured cardiac myocytes in response to hypoxia/reoxygenation with an anti-eIF5A

antibody (J-M) and protein A-labelled colloidal gold.

Supplementary Figure 5: Expression of eIF5A in myocardial tissues subjected to ischemia/reperfusion *in vivo*. Immunofluorescence localization of eIF5A protein in myocardial tissues from sham-operated rats and rats subjected to myocardial ischemia/reperfusion *in vivo*. The procedures for myocardial ischemia/reperfusion were as described elsewhere[36].

Supplementary Figure 6: Secreted-re-eIF5A (10 μg/ml) induced phosphorylation of ERK1/2, SAPKs , p38MAPK, IκB, and ATF2 in cultured rat cardiac myocytes. The procedures for Western analysis were as described previously[9]. The experiments were performed at least in triplicate. The results shown are from one representative experiment.

Supplementary Figure 7: Effects of secreted re-eIF5A (10 μg/ml) on phospho-kinase activation in human skeletal muscle myoblasts (ZenBio, Inc.). Cell lysates were applied to Human Phospho-Kinase Array (R & D Systems, Inc.; according to the manufacturer's instructions).

Supplementary Figure 8: Secreted re-eIF5A increased phosphorylation of ATM and p53. (a) Secreted re-eIF5A (10 μg/ml) increased phosphorylation of ATM at Ser1981 (10H11.E12; Cell Signaling Technology) and p53 at Ser15 (#9284; Cell Signaling) and Ser20 (sc-18078; Santa Cruz), but not Ser46 (ab76242; Abcam). The experiments were performed at least in triplicate. The results shown are from one representative experiment. Control antibodies were used for ATM (sc-7230; Santa Cruz) and p53 (sc-55476; Santa Cruz). (b) and (c) Effects of a specific p53 inhibitor, pifithrin-α (30 μM, pretreated for 24 h) on apoptosis induction of cardiac myocytes by secreted re-eIF5A (10 μg/ml, 48 h) as determined by TUNEL staining (brown) and cardiac myosin immunostaining (blue) as in Fig. 3a. * P <0.0001 vs. secreted re-eIF5A only (mean ± s.e.m., n=4 for each) (Dunnett's multiple comparison test).

Supplementary Figure 9: Effects of PARP-1 inhibitor (3-aminobenzamide [Sigma], 2 mM) or caspase inhibitor (Z-VAD.fmk [BIOMOL International, LP], 100 μM), added 1 h before addition of secreted-re-eIF5A (10 μg/ml), on the induction of apoptosis in cardiac myocytes. (a) TUNEL staining (brown) and cardiac myosin immunostaining (blue) as in Fig. 3a. Representative images at 72 h after addition of secreted-re-eIF5A. (b) The percentage of apoptotic cardiac myocytes determined by TUNEL staining at 72 h after addition of secreted re-eIF5A. Data are expressed as (mean ± s.e.m., n=4 for each). *$P < 0.0001$ vs. secreted re-eIF5A only (Dunnett's multiple comparison test).

Supplementary Figure 10: Effects of neutralization with anti-eIF5A mAbs (0.02 mg/ml) on hypoxia (60 min)/reoxygenation (15 min)-induced activation of ERK1/2 in cultured cardiac myocytes. * P <0.001 vs. Unstimulated ** P <0.01 and *** P <0.05 vs. mouse IgG (mean ± s.e.m., n=4 for each) (Dunnett's multiple comparison test).

Supplementary Figure 11: Effects of neutralization with anti-eIF5A mAbs (0.02 mg/ml) on hypoxia (11 h)/reoxygenation (48 h)-induced cytosolic release of cytochrome *c* (a) as well as caspase-3 activation (b) in cultured cardiac myocytes. (a) * P <0.0002 vs. Unstimulated ** P <0.0002 and *** P <0.0005 vs. mouse IgG (mean ± s.e.m., n=4 each). (b) * P <0.0001 vs. Unstimulated ** P <0.001 vs. mouse IgG (mean ± s.e.m., n=4 for each) (Dunnett's multiple comparison test).

Supplementary Figure 12: Effects of neutralization with anti-eIF5A mAbs (0.02 mg/ml) on hypoxia (15 h)/reoxygenation (72 h)-induced apoptosis induction in cultured cardiac myocytes. (a) TUNEL staining (brown) and cardiac myosin immunostaining (blue) as in Fig. 3a. Representative images at 72 h after reoxygenation. (b) The percentage of apoptotic cardiac myocytes determined by TUNEL staining at 72 h after reoxygenation. * P <0.0001 vs. Unstimulated ** P <0.0001 and *** P <0.001 vs. Mouse IgG (mean ± s.e.m., n=4 for each) (Dunnett's multiple comparison test).

Supplementary Figure 13: Effect of neutralization with an anti-eIF5A mAb (YSP5-45-36; 0.02 mg/ml) on UV irradiation (100 mJ/cm^2, 15 min; UV lamp, FL20S E-30/DMR, peak wave 305 nm, Toshiba Medical Supply, Tokyo)-induced activation of ERK1/2 in cultured cardiac myocytes. * P <0.0001 vs. Unstimulated ** P <0.001 vs. Mouse IgG (mean ± s.e.m., n=4 for each) (Dunnett's multiple comparison test).

Supplementary Figure 14: Effects of neutralization with an anti-eIF5A mAb (YSP5-45-36; 0.02 mg/ml) on UV irradiation (100 mJ/cm^2)-induced cytosolic release of cytochrome *c* (a) as well as caspase-3 activation (b) in cultured cardiac myocytes. (a) * P <0.0001 vs. Unstimulated ** P =0.0001 vs. Mouse IgG (mean ± s.e.m., n=4 for each). (b) * P =0.0001 vs. Unstimulated, ** P <0.0003 vs. Mouse IgG (mean ± s.e.m., n=4 for each) (Dunnett's multiple comparison test).

Supplementary Figure 15: Effect of neutralization with an anti-eIF5A mAb (YSP5-45-36; 0.02 mg/ml) on UV irradiation (100 mJ/cm^2)-induced apoptosis induction of cultured cardiac myocytes. (a) TUNEL staining (brown) and cardiac myosin immunostaining (blue) as in Fig. 3a. Representative images at 72 h after reoxygenation. (b) The percentage of apoptotic cardiac myocytes determined by TUNEL staining at 72 h after reoxygenation. * P <0.0001 vs. Unstimulated **P<0.0001 vs. Mouse IgG (mean ± s.e.m., n=4 for each) (Dunnett's multiple comparison test).

Supplementary Figure 16: Standard curve of the sandwich enzyme-linked immunosorbent assay (ELISA) for eIF5A. The sandwich ELISA was performed with YSPN2-74-18 (20 μg/ml) as a capture antibody fixed on the wells of microtiter strips. Plasma samples and standards of known human re-eIF5A were pipetted into the wells and incubated. After washing, horseradish peroxydase (HRP)-labeled YSP5-45-36 (1 μg/ml) was added as a detection antibody and incubated. After washing, color development was carried by addition of a substrate solution. Values are means ± s.d., (n=4).

Supplementary Figure 17: Plasma levels of eIF5A in rats with heart subjected to UV-irradiation (1 J/cm^2) *in vivo*. After UV-irradiation, plasma eIF5A levels significantly increased and reached the peak levels (507.0 ± 102.5 ng/ml [mean ± s.e.m.]) at 5 to 20 min as compared with control levels (49.7 ± 13.0 ng/ml; n=10, *P*=0.0007). *P* value was calculated by paired *t*-test.

Supplementary Figure 18: Effects of *in vivo* treatment with either anti-eIF5A neutralizing mAbs (YSP5-45-36 [a], YSPN2-74-18 [b]) at various doses on rat myocardial ischemia/reperfusion injury. (a) *p<0.0001 and **P<0.02 vs. Mouse IgG (mean ± s.e.m., n=12, for each group). (b) *p<0.0001, **P<0.001, and ***P<0.01 vs. Mouse IgG (mean ± s.e.m., n=12, for each group) (Dunnett's multiple comparison test).

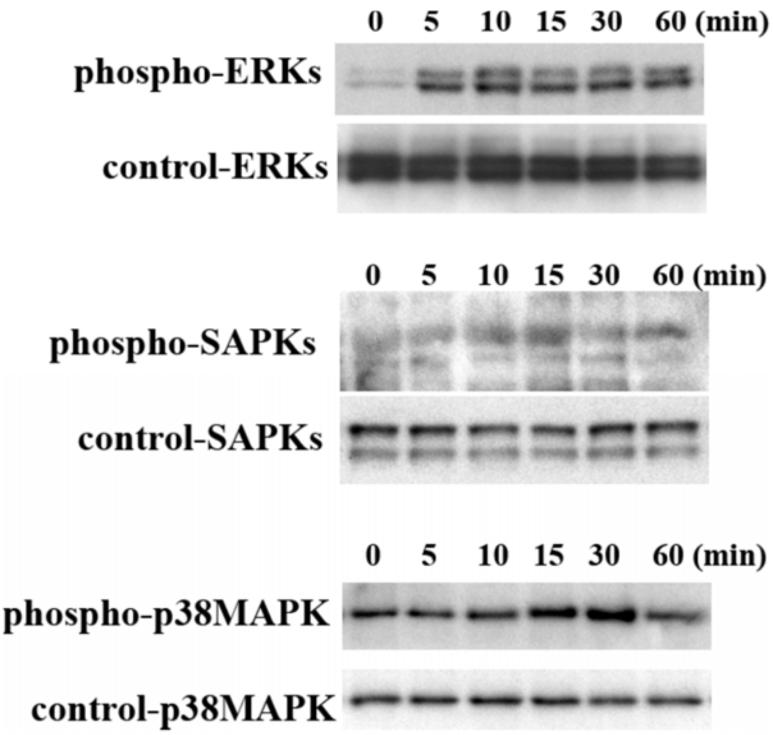

Supplementary Figure 1

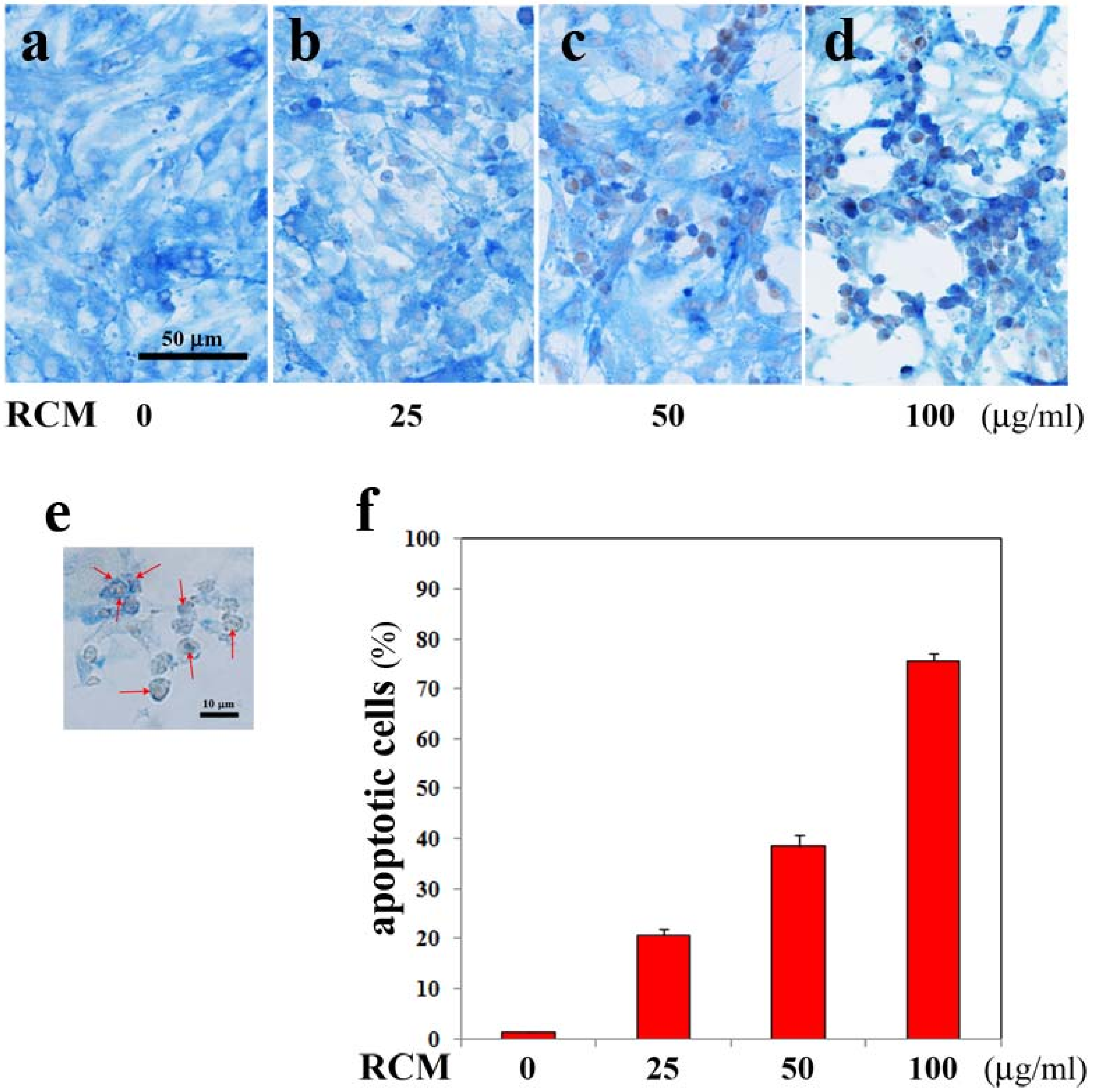

Supplementary Figure 2

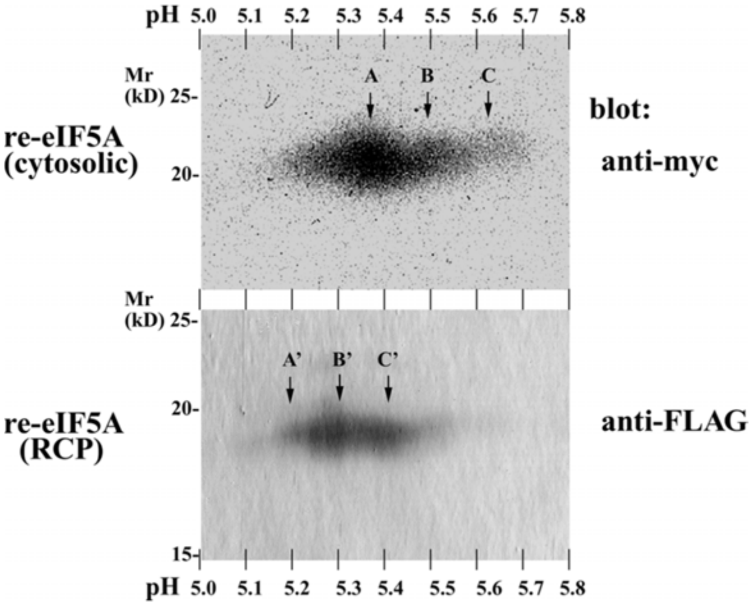

Supplementary Figure 3

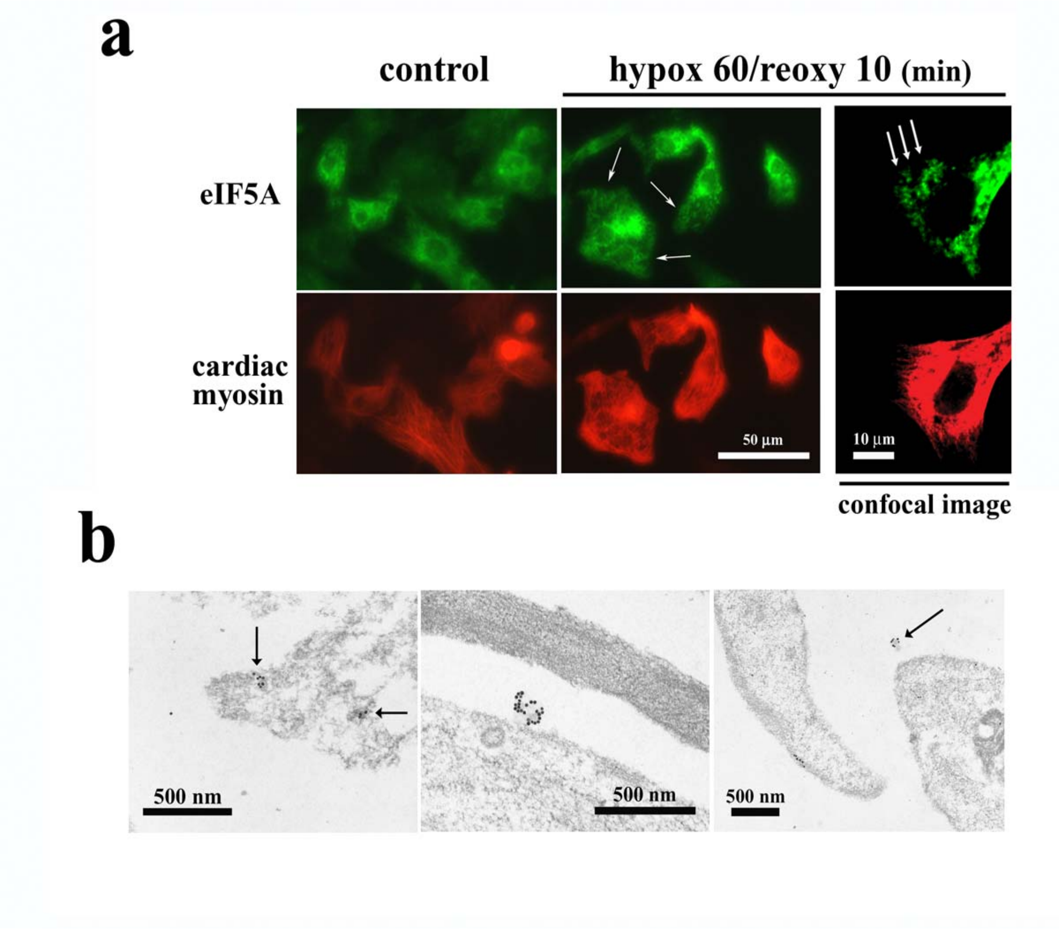

Supplementary Figure 4

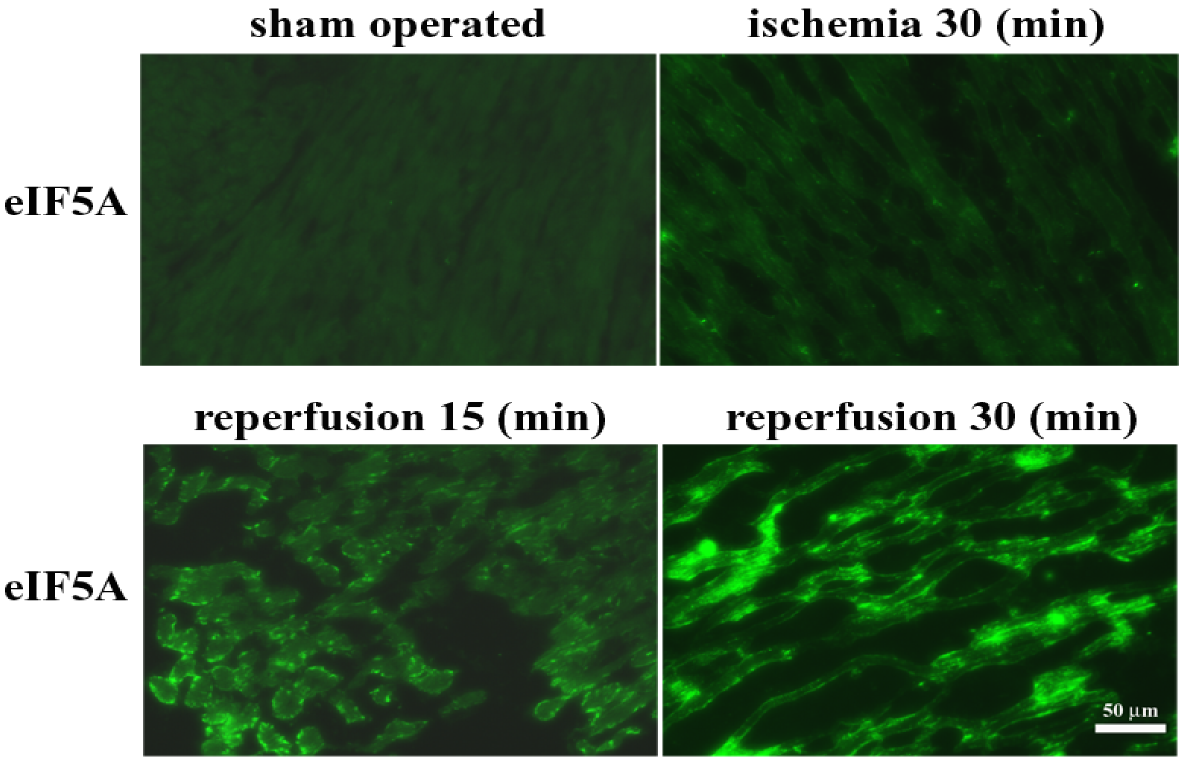

Supplementary Figure 5

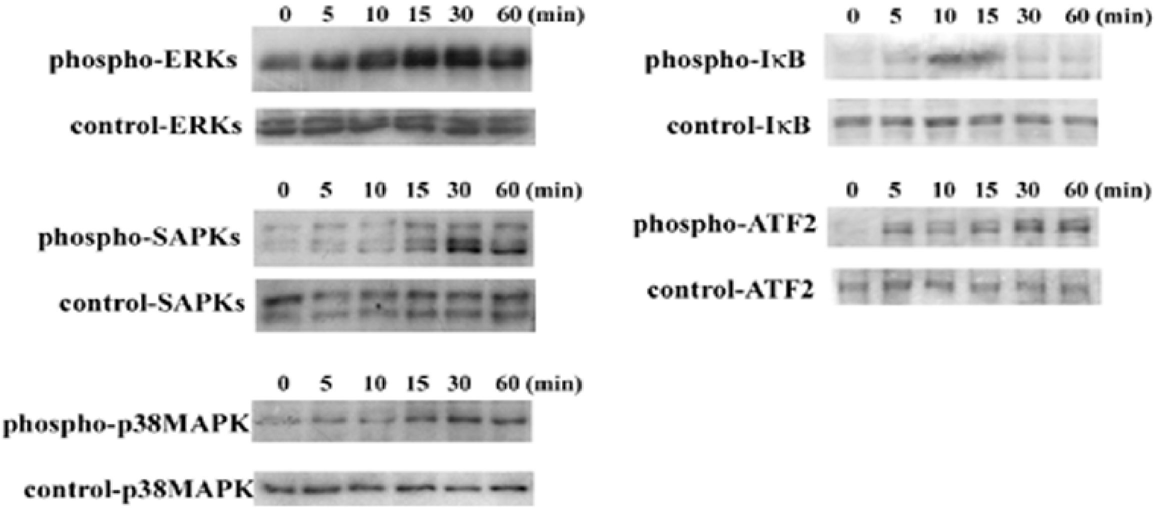

Supplementary Figure 6

a

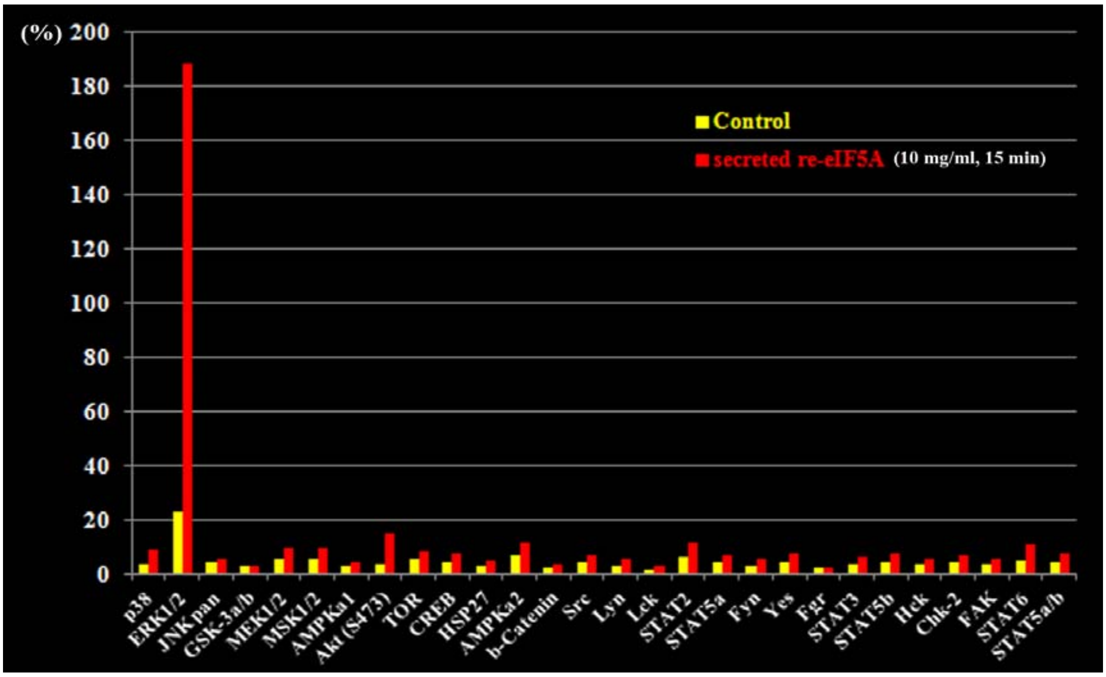

b

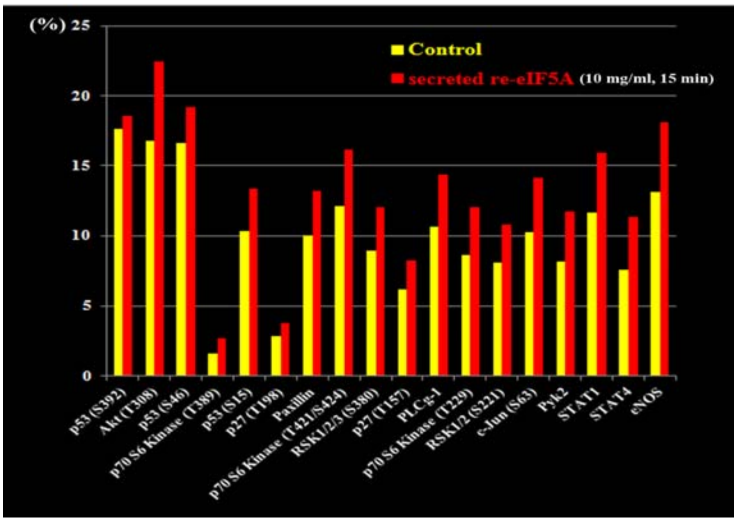

Supplementary Figure 7

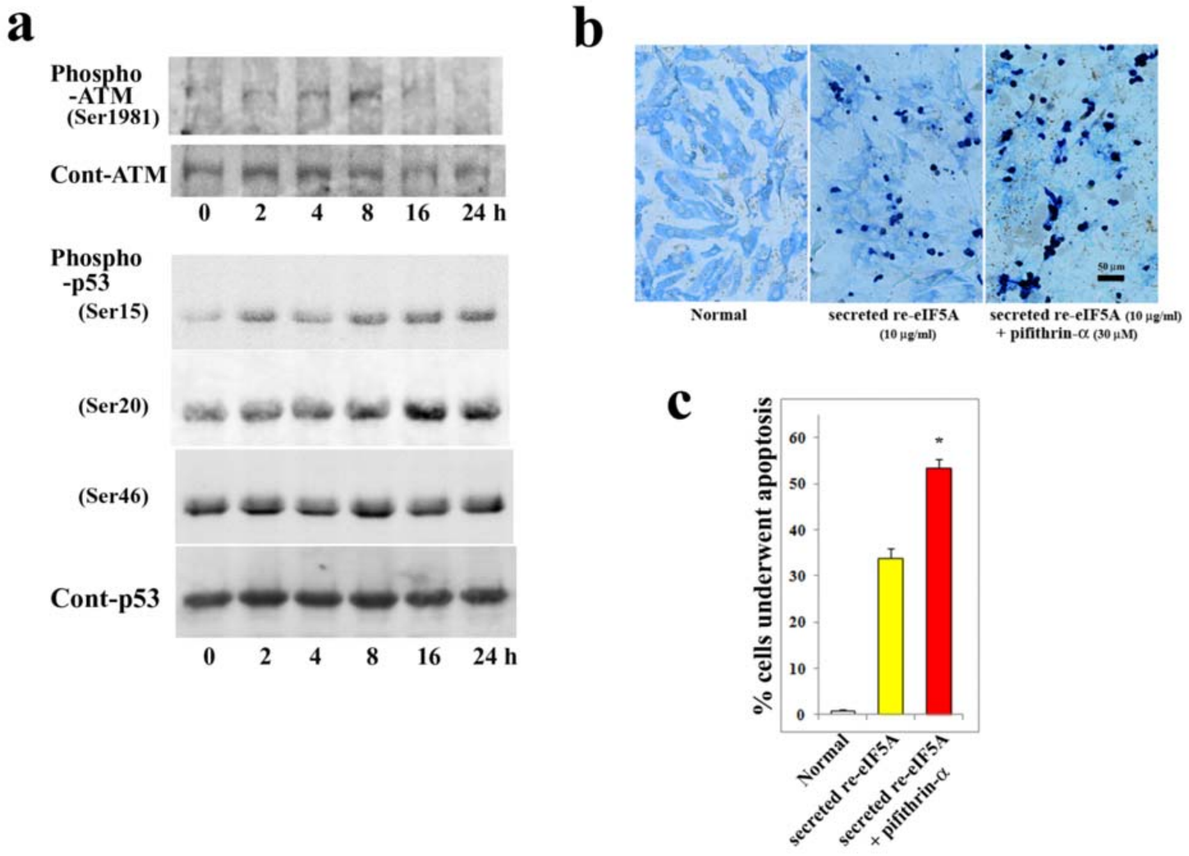

Supplementary Figure 8

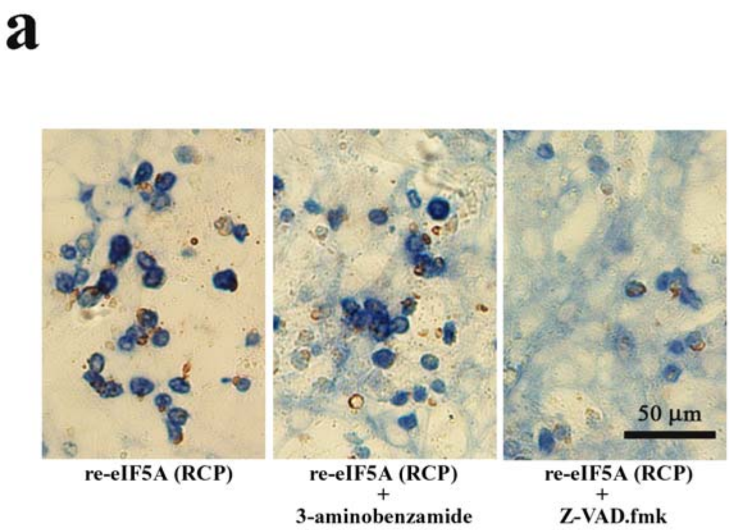

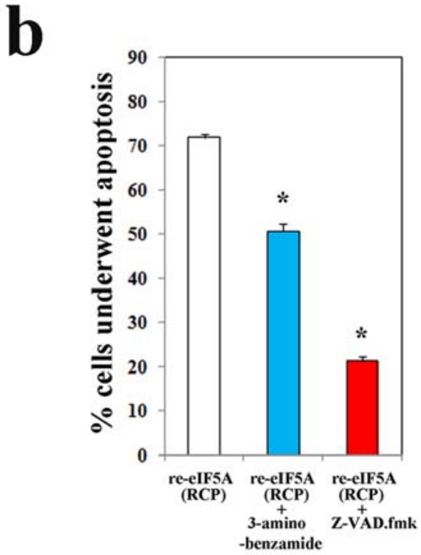

Supplementary Figure 9

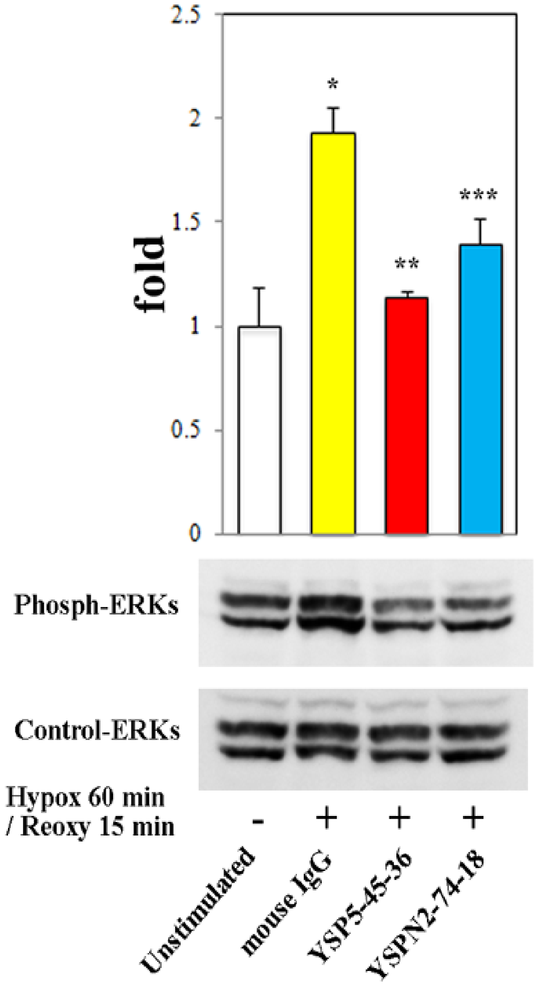

Supplmentary Figure 10

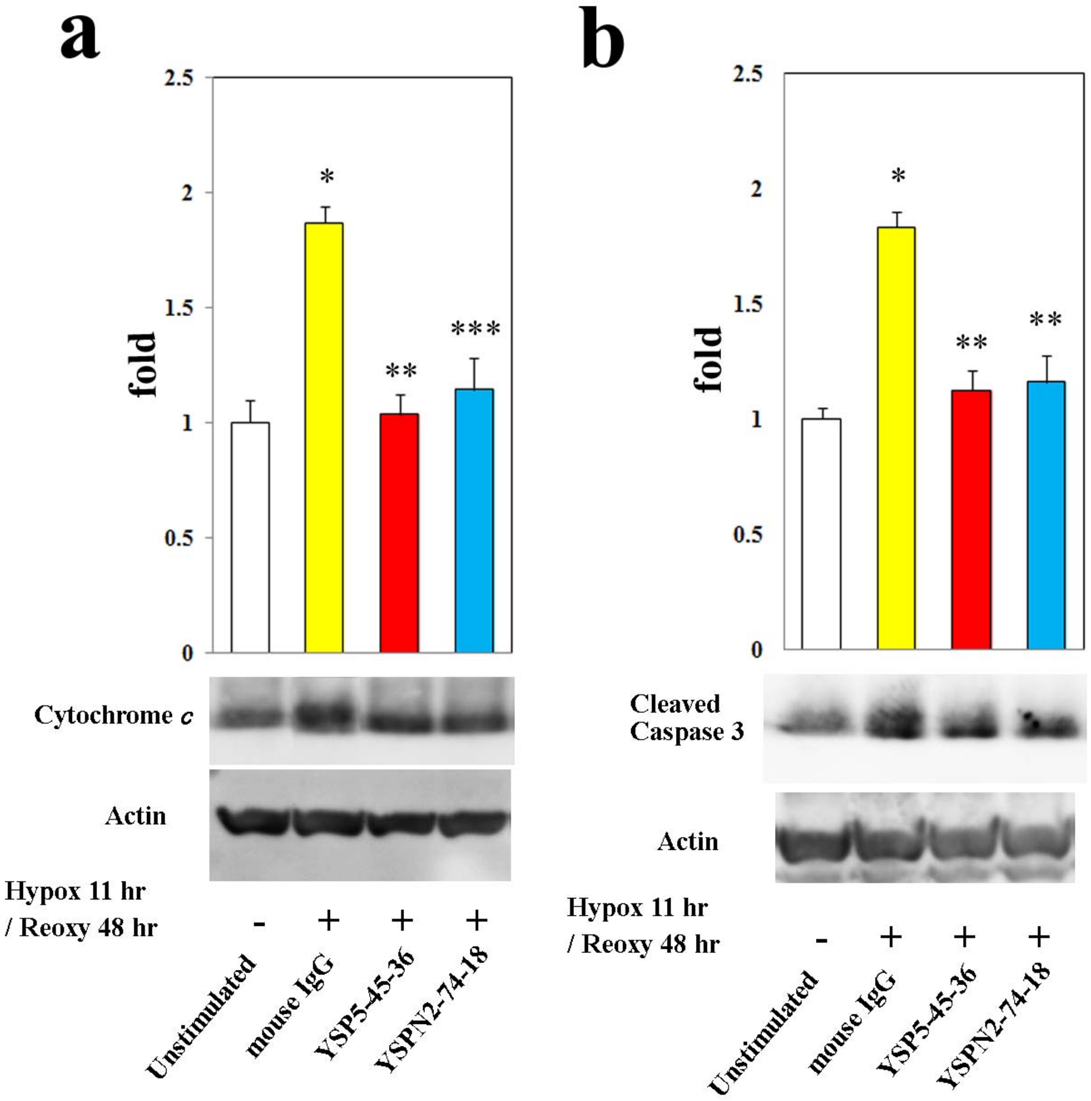

Supplmentary Figure 11

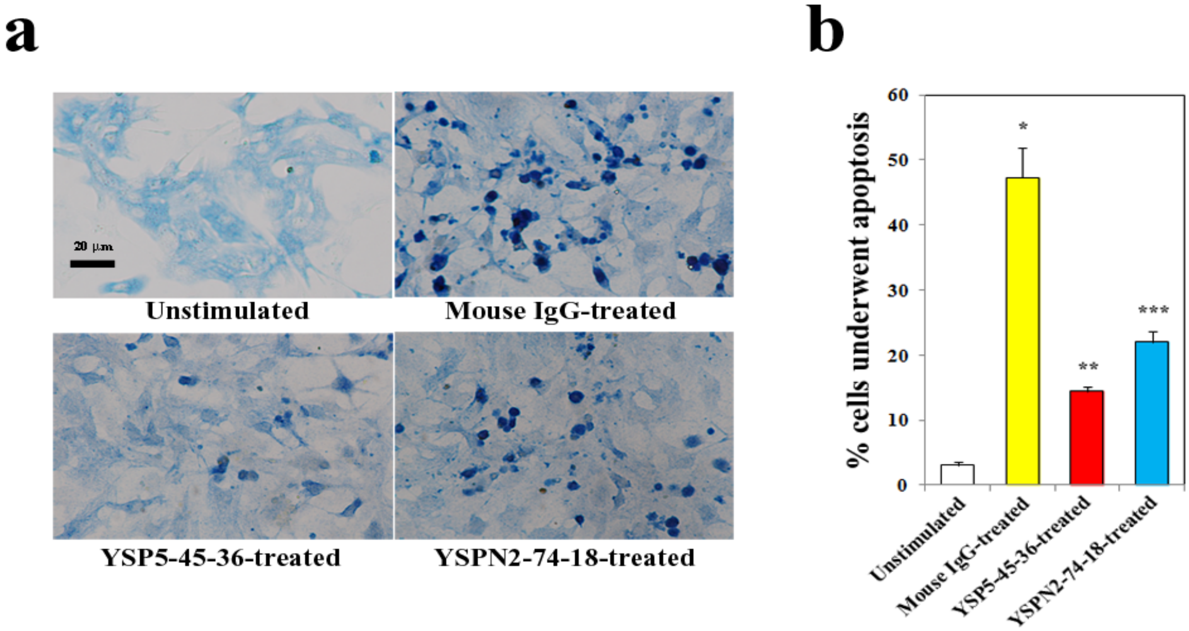

Supplmentary Figure 12

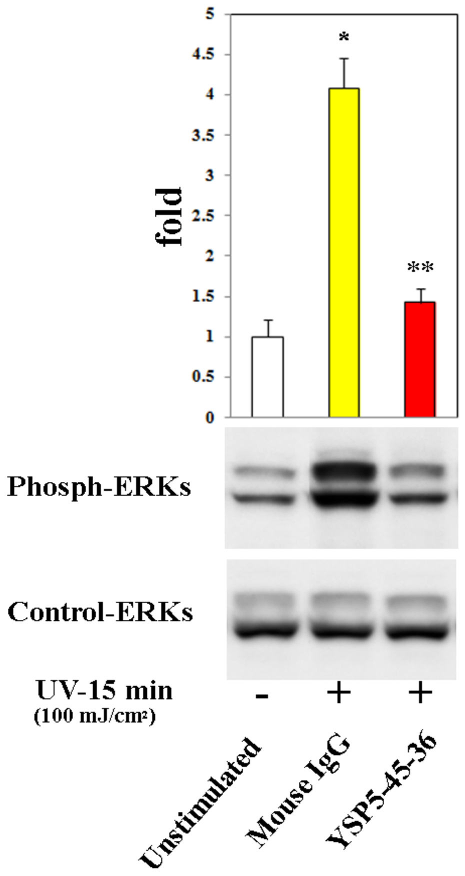

Supplmentary Figure 13

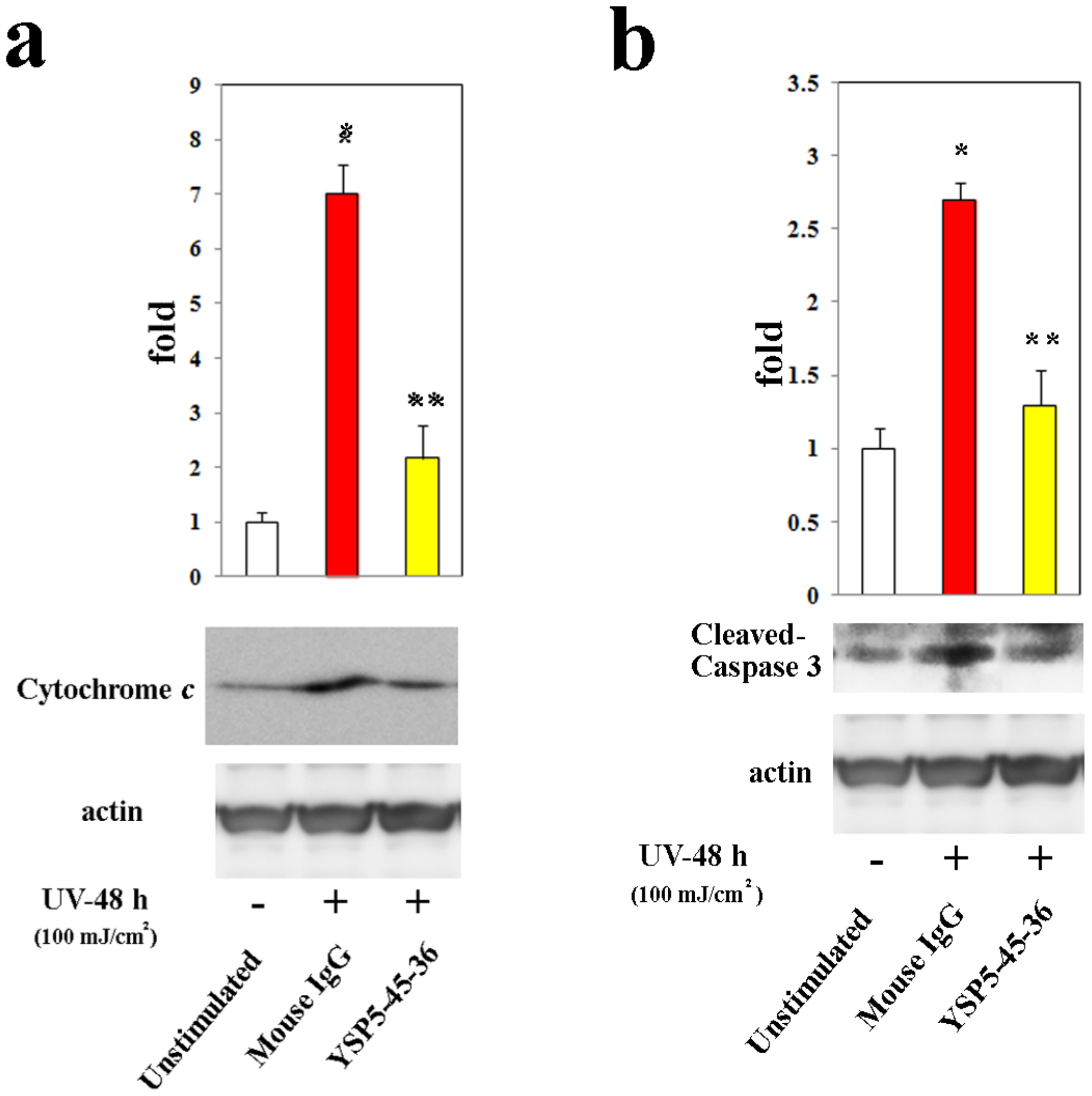

Supplmentary Figure 14

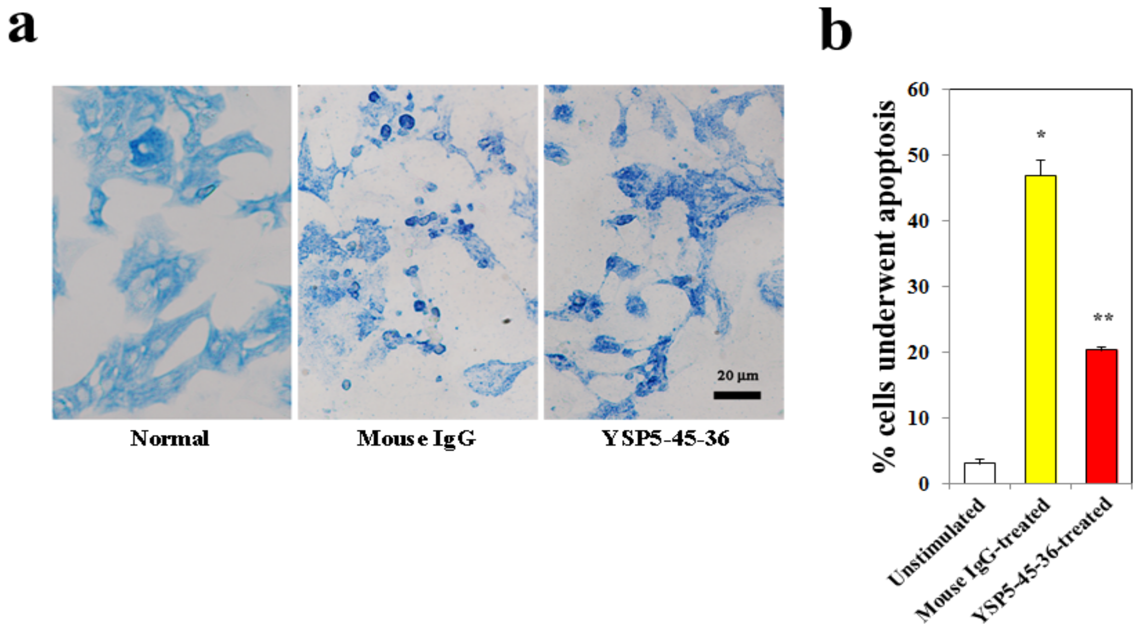

Supplmentary Figure 15

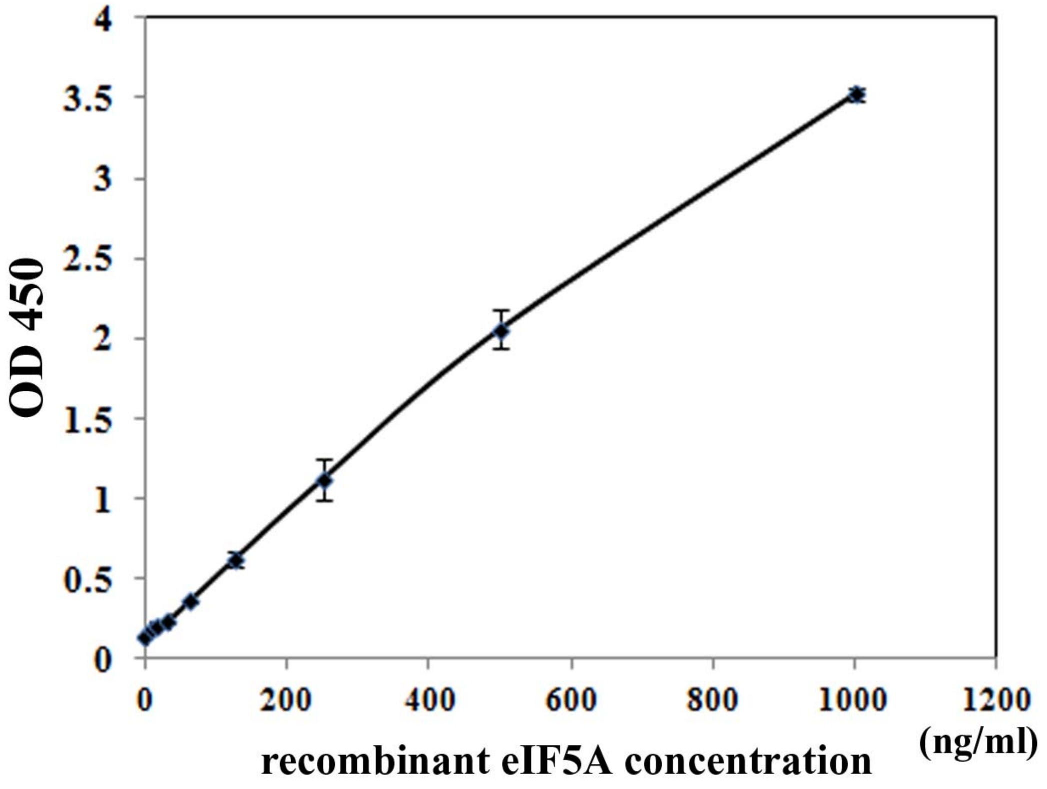

Supplmentary Figure 16

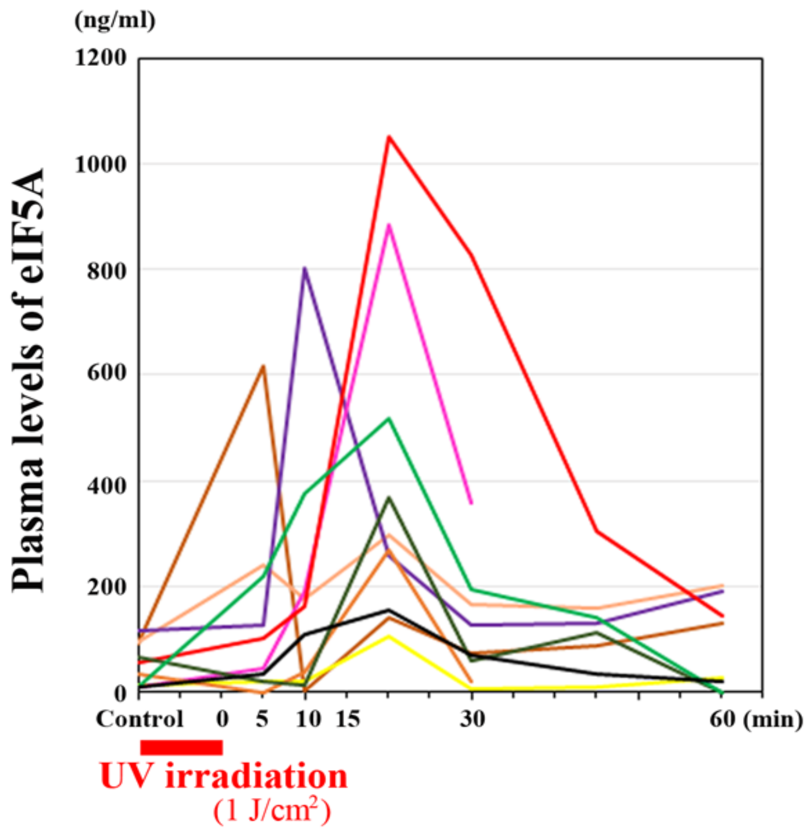

Supplmentary Figure 17

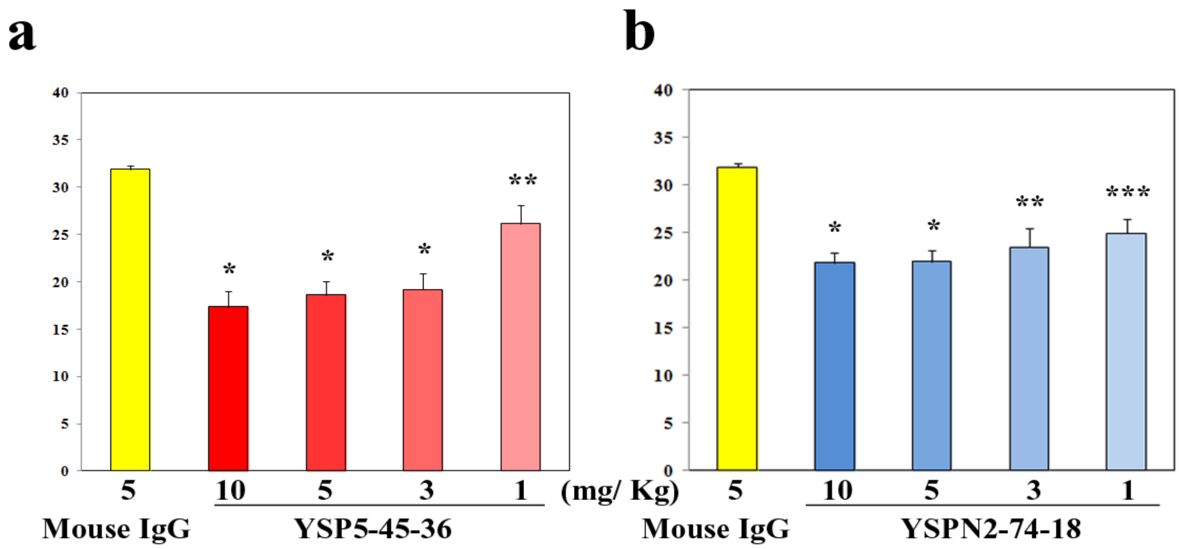

Supplmentary Figure 18
